# Single-cell analysis reveals the chemotherapy-induced cellular reprogramming and novel therapeutic targets in relapsed/refractory acute myeloid leukemia

**DOI:** 10.1038/s41375-022-01789-6

**Published:** 2022-12-21

**Authors:** Kening Li, Yuxin Du, Yun Cai, Wenjie Liu, Yan Lv, Bin Huang, Lishen Zhang, Zhi Wang, Ping Liu, Qian Sun, Ning Li, Mengyan Zhu, Bakwatanisa Bosco, Liangyu Li, Wei Wu, Lingxiang Wu, Jianyong Li, Qianghu Wang, Ming Hong, Sixuan Qian

**Affiliations:** 1 The Affiliated Cancer Hospital of Nanjing Medical University, Jiangsu Cancer Hospital, Jiangsu Institute of Cancer Research, 210002 Nanjing, China; 2 Center for Global Health, School of Public Health, Nanjing Medical University, 211166 Nanjing, Jiangsu China; 3 Department of Bioinformatics, Nanjing Medical University, 211166 Nanjing, China; 4 Department of Hematology, The First Affiliated Hospital of Nanjing Medical University, Jiangsu Province Hospital, Nanjing, 210029 China; 5 Key Laboratory of Hematology of Nanjing Medical University, Nanjing, 210029 China; 6 Department of Hematology, The Affiliated Wuxi No. 2 People’s Hospital of Nanjing Medical University, Wuxi, 214000 Jiangsu China; 7 Pukou CLL Center, Nanjing, 210000 China; 8 Jiangsu Key Lab of Cancer Biomarkers, Prevention and Treatment, Collaborative Innovation Center for Personalized Cancer Medicine, Nanjing Medical University, Nanjing, Jiangsu 211166 China; 9 Biomedical Big Data Center, Nanjing Medical University, Nanjing, Jiangsu 211166 China

**Keywords:** Cell biology, Acute myeloid leukaemia

## Abstract

Chemoresistance and relapse are the leading cause of AML-related deaths. Utilizing single-cell RNA sequencing (scRNA-seq), we dissected the cellular states of bone marrow samples from primary refractory or short-term relapsed AML patients and defined the transcriptional intratumoral heterogeneity. We found that compared to proliferating stem/progenitor-like cells (PSPs), a subpopulation of quiescent stem-like cells (QSCs) were involved in the chemoresistance and poor outcomes of AML. By performing longitudinal scRNA-seq analyses, we demonstrated that PSPs were reprogrammed to obtain a QSC-like expression pattern during chemotherapy in refractory AML patients, characterized by the upregulation of CD52 and LGALS1 expression. Flow cytometric analysis further confirmed that the preexisting CD99^+^CD49d^+^CD52^+^Galectin-1^+^ (QSCs) cells at diagnosis were associated with chemoresistance, and these cells were further enriched in the residual AML cells of refractory patients. Interaction of CD52-SIGLEC10 between QSCs and monocytes may contribute to immune evading and poor outcomes. Furthermore, we identified that LGALS1 was a promising target for chemoresistant AML, and LGALS1 inhibitor could help eliminate QSCs and enhance the chemotherapy in patient-derived primary AML cells, cell lines, and AML xenograft models. Our results will facilitate a better understanding of the AML chemoresistance mechanism and the development of novel therapeutic strategies for relapsed/refractory AML patients.

## Introduction

Acute myeloid leukemia (AML) is the most common and lethal adult acute leukemia, characterized by aggressive proliferation, differentiation blockage, and apoptosis disorder of immature blasts in the bone marrow, peripheral blood, and other tissues [1]. Chemotherapy is the primary treatment for AML [2]. However, approximately 10–40% of newly diagnosed AML patients do not achieve complete remission (CR) with initial treatment and are categorized as primary refractory [3]. Besides, more than half of the patients who initially achieve CR will eventually relapse [4]. Chemoresistance and relapse are the leading cause of AML-related deaths.

The high intratumoral heterogeneity (ITH) is one of the most important reasons for drug resistance and relapse in AML [5, 6]. Although many previous studies suggested that leukemia stem cells (LSCs) contributed to the chemoresistance and poor outcome of AML [7–12], other studies have reported contrary observations [13, 14]. These controversial conclusions in the field may result from the fact that LSC is a highly heterogeneous cell group consisting of subgroups with different drug sensitivity [15–18], but previous studies based on bulk sequencing only obtain the average expression level of various cellular states, thus failing to capture the specific characteristics of cell subpopulations.

The emergence of single-cell sequencing technologies makes it possible to quantify the whole genome or transcriptome of every single cell in a tissue mixture and provides an unprecedented opportunity to decipher the complexity of AML cellular heterogeneity [9, 19–21]. For example, some recent studies provide important references for understanding the transcriptomic ITH in diagnostic AML by utilizing scRNA-seq [19, 21], but the chemoresistant LSC subpopulations in refractory or short-term relapsed AML patients remain unclear. Despite several single-cell genomic analyses having deciphered the clonal evolutionary process of therapeutic resistance at the cellular subpopulation level [22–25], the chemotherapy-induced plasticity of cellular states and transcriptomic reprogramming, as well as the molecular interactions between chemoresistant LSCs and microenvironment are yet understudied currently [15]. In addition, the strategies for targeting chemoresistant LSC subpopulations are still needed to be further identified. Identifying the intrinsic resistant LSC subpopulation and deciphering the cellular reprogramming induced by chemotherapy in relapsed/refractory AML (RR-AML), will help to understand the underlying mechanism of chemoresistance and provide potential therapeutic strategies for targeting the chemoresistant LSC subpopulation.

Here, we performed an integrated single-cell transcriptomic analysis of bone marrow samples from primary refractory and short-term relapsed AML patients to identify the critical factors in AML chemoresistance. Besides, longitudinal scRNA-seq analysis was performed to trace the dynamic cellular and molecular plasticity induced by chemotherapy in AML patients. By comprehensively dissecting the cellular states of leukemia-like cells in the AML ecosystem, we defined the transcriptional ITH and identified the LSC subpopulation that was involved in the chemoresistance and relapse of AML. Furthermore, the reprogramming of cellular states induced by chemotherapy was described and effective therapeutic targets for chemotherapy sensitization were identified for RR-AML patients.

## Results

### Identifying the leukemia-like cells by integrating scRNA-seq data from healthy and RR-AML bone marrow samples

**Fig. 1:**
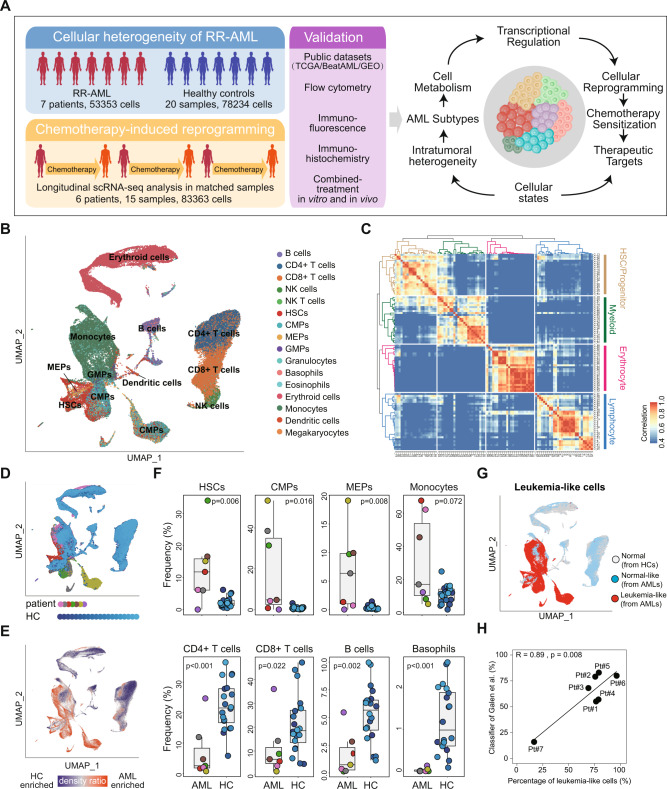
Integrated scRNA-seq analysis of healthy and RR-AML bone marrow samples. **A** Schematic overview and the number of samples and cells in this study. The cellular heterogeneity of RR-AML patients was deconstructed by integrating scRNA-seq data of seven RR-AML patients (in-house) and 20 healthy controls (GSE120221). Chemotherapy-induced reprogramming was investigated by analyzing longitudinal scRNA-seq data of 15 samples from six patients (in-house dataset and the public GSE116256 data). Results derived from scRNA-seq data were further validated by bulk transcriptomic data and in vitro and in vivo experiments. **B** UMAP visualization of cell type identification based on SingleR. **C** Heatmap showing the correlation of 98 clusters. Four major cell types were identified by unsupervised clustering, including HSC/Progenitors (brown), myeloid cells (green), erythrocytes (pink), and lymphocytes (blue). **D** UMAP visualization of 118,565 single cells that passed quality controls, colored by seven RR_AML patients and twenty HCs. **E** Heatmap showing the density ratio of the UMAP projections of RR-AML and healthy bone marrow cells. The UMAP visualization is split into 400 × 400 bins. **F** Boxplot showing the proportion of cell types in RR-AML patients and HCs. Wilcoxon rank-sum test was used to measure the differences between the two groups. **G** UMAP visualization of the identified leukemia-like cells. Cells from HCs were shown in gray, normal-like cells from AML patients were shown in sky blue, and leukemia-like cells from AML patients were shown in red. **H** Scatterplot showing the correlation between the proportions of leukemia-like cells predicted by this study (*x*-axis) and the classifier of Galen et al. (*y*-axis).

To explore the ITH and identify potential therapeutic targets for RR-AML, we designed a scRNA-seq-based workflow to comprehensively dissect the dynamic cellular and molecular reprogramming during chemotherapy in RR-AML patients (Fig. 1A). Firstly, we performed scRNA-seq on bone marrow specimens from seven AML patients who were short-term relapsed or primary refractory after cytarabine-based chemotherapy (Supplementary Table S1). Four of these patients have received at least two courses of induction treatment but still have more than 20% bone marrow blasts, and the other three patients were short-term relapsed (<6 months) after achieving CR (Supplementary Fig. S1A). After quality control, we got transcriptomic data of 4426~11,767 single cells for each patient.

Considering the similarities of differentiation hierarchies between leukemia and normal cells in the AML ecosystem [19], we integrated bone marrow scRNA-seq data from 20 healthy controls (HCs; GSE120221) [26] and seven AML patients to compare the differences between physiological and pathological states and distinguish the leukemia-like cells from normal-like cells in AML patients. Totally 118,565 cells were integrated after quality control, including 44,151 cells from AML patients and 74,414 cells from healthy donors. We performed unsupervised clustering of all these cells at high resolution and identified 98 transcriptional cell clusters (Supplementary Fig. S1B). Hierarchical clustering, SingleR analysis [27], and the expression of known markers suggested that these clusters could be classified into various cell types, including hematopoietic stem cells (HSCs), common myeloid progenitor (CMP), megakaryocyte-erythroid progenitor (MEP), granulocyte-monocyte progenitor (GMP), dendritic cells (DCs), monocytes, erythrocytes, T cells, B cells, and natural killer (NK; Fig. 1B, C and Supplementary Fig. S1C). Compared with HCs, the cells from AML patients were enriched in primitive and myeloid cells (Fig. 1D, E), such as HSC, MEP, CMP, and monocytes. In contrast, the lymphocytes were depleted in AML patients, such as B cells, CD4^+^ T cells, and CD8^+^ T cells (Fig. 1F). Moreover, results showed that cells from AML patients had significantly higher proliferation and stemness scores than cells from HCs (Wilcoxon rank-sum test, *p* < 2.2e–16; Supplementary Fig. S1D–G).

According to the proportion of cells from AML patients in each transcriptional cluster, we distinguished the leukemia-like cells from normal-like cells in the ecosystem of AML (Fig. 1G; Supplementary Methods). Our predictive percentage of leukemia-like cells was highly accordant with the results of the classifier from Galen et al. [19] (Pearson *r* = 0.89, *p* = 0.008; Fig. 1H and Supplementary Fig. S1H), which could identify the leukemia-like cells by taking genetic mutation into consideration. In addition, to further validate the identified leukemia-like cells, we compared the proliferation and stemness scores between the leukemia-like and normal-like cells in the AML bone marrow. Results showed that these scores were significantly higher in leukemia-like cells than in normal-like cells (Supplementary Fig. S1I, J). Besides, the scores of LSC signatures reported by previous studies were also remarkably higher in leukemia-like cells than in normal-like cells, such as the LSC_up and LSC17 signatures by Eppert et al. [28] and Ng et al. [29] (Supplementary Fig. S1K), indicating the reliability of the identification of leukemia-like cells based on the transcriptomic data at the single-cell level.

### The transcriptional ITH is associated with AML outcomes

**Fig. 2:**
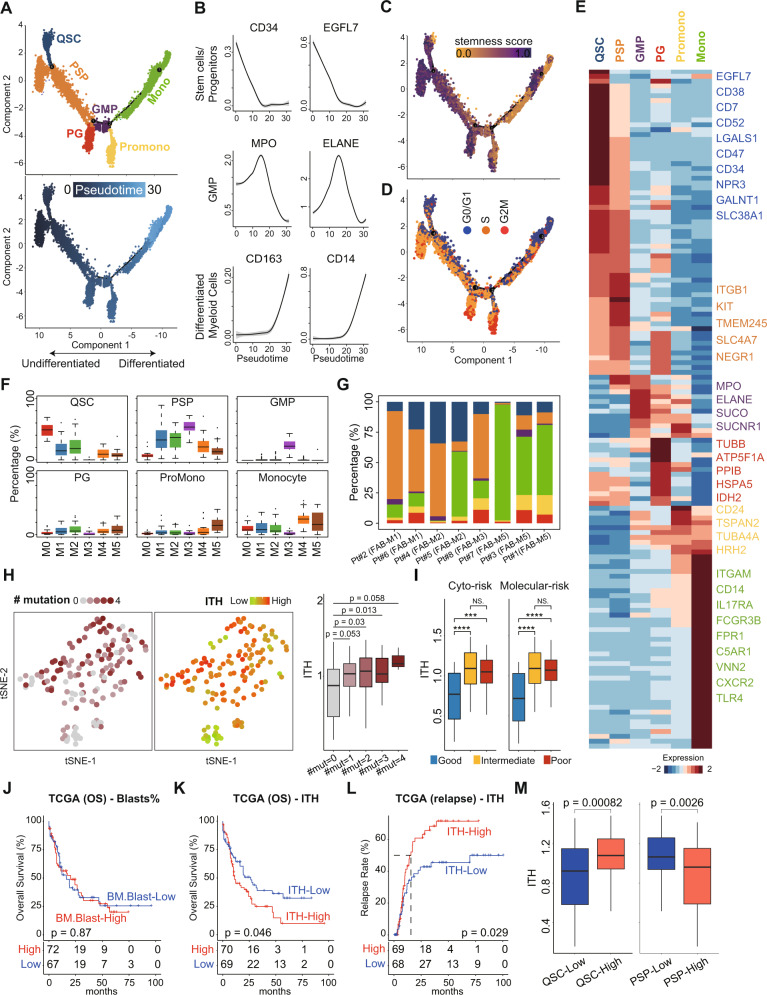
Dissection of transcriptional ITH of RR-AML bone marrow. **A** Pseudotime trajectory of leukemia-like cells. Cellular states are defined based on differentiation stages, proliferation, and stemness scores. **B** Expression of well-defined lineage-specific genes along with the pseudotime trajectory. The curves were fitted by locally weighted regression (LOESS). **C** The stemness score calculated by CytoTRACE. **D** The cell cycle phases of all leukemia-like cells. **E** Cell surface markers that are specifically expressed on each cellular state. **F** Boxplots showing the percentage of each cellular state in TCGA AML patients with different FAB subtypes based on the estimate of CIBERSORTx. **G** The percentage of each cellular state in leukemia-like cells of AML patients with different FAB subtypes at the single-cell level. **H** Left: t-SNE plots showing the number of mutated leukemia-associated genes and ITH scores in the TCGA AML patients. Right: Boxplot showing the ITH of TCGA AML patients with the different number of mutated genes. Wilcoxon rank-sum test was used to measure the differences between groups. **I** Boxplot showing the ITH of AML patients with different cytogenetic and molecular risks. Wilcoxon rank-sum test was used to measure the differences between groups. **J** Kaplan–Meier analysis of overall survival (OS) of TCGA AML patients. All patients were categorized into two groups based on the median of blast percentage in the bone marrow. **K**, **L** Kaplan–Meier analysis of OS (**K**) and relapse (**L**) of TCGA AML patients. All patients were categorized into two groups based on the median of the ITH score. **M** The ITH scores of TCGA AML patients with different levels of QSCs (left) and PSPs (right). Patients were categorized into two groups based on the median of QSCs or PSPs abundance.

We next attempted to deconstruct the cellular diversity of these leukemia-like cells. Pseudotime analysis revealed that leukemia-like cells showed a differentiation trajectory similar to normal hematopoietic development (Fig. 2A). According to the expression of established lineage markers (Fig. 2B and Supplementary Fig. S2A), stemness score (Fig. 2C) and proliferation status (Fig. 2D), we identified six types of leukemia-like cellular states, including quiescent stem-like cells (QSCs), proliferating stem/progenitor-like cells (PSPs), GMP, proliferating granulocyte (PG), promonocytes (hereinafter referred to as promono), and differentiated monocytes (hereinafter referred to as mono). The specific genes highly expressed in each cellular state were shown in Supplementary Table S2. Based on the information in Cell Surface Protein Atlas (CSPA) [30] and Gene Ontology (GO: 0009986), we identified the specifically expressed cell surface markers in each state (Fig. 2E). Results showed that CD52, LGALS1, and CD47 were upregulated in QSCs, compared with other cellular states. Notably, both the QSCs and PSPs have significantly higher LSC-related scores than other leukemia cells (Supplementary Fig. S2B), indicating that the heterogeneity of LSCs could be dissected by single-cell transcriptomic analysis. Although QSCs and PSPs had comparable stemness, these two primitive subpopulations exhibit different proliferation features (Supplementary Fig. S2C). Gene Set Enrichment Analysis (GSEA) further validated that QSCs highly expressed the quiescence signature (NES = 1.95, *p* = 0.0015), while PSPs enriched in the cell dividing signature (NES = –1.99, *p* = 0.0033; Supplementary Fig. S2D).

Using our single-cell transcriptomic profile as a reference, we dissected the cellular components of each TCGA sample by utilizing CIBERSORTx [31]. Results showed that the composition of cellular states in AML patients was extensively varied. Some patients had one dominant cellular state, while other patients contained two or more cellular states (Supplementary Fig. S2E). The cellular components showed association with the FAB subtypes of AML patients (Fig. 2F). The QSCs were mainly enriched in the FAB-M0 and intermediately enriched in the FAB-M1 and FAB-M2 AML. The PSPs were highly enriched in FAB-M1, FAB-M2 and acute promyelocytic leukemia (APL, FAB-M3). The GMPs were mainly enriched in APL. The promono and mono had the highest level in FAB-M4 and FAB-M5, with an intermediate level in other subtypes. This association was also validated in two independent cohorts (Supplementary Fig. S2F, G). In addition, these observations were further proved at the single-cell level by showing the cellular components in AML patients of different subtypes (Fig. 2G and Supplementary Fig. S2H), indicating that the cellular components were associated with the previously established subtyping of AML.

Using Shannon entropy (see Supplementary Methods), we measured the transcriptional ITH of each patient based on the identified cellular states. Results showed that patients who harbored more AML-related mutations had a higher transcriptional ITH score (Fig. 2H), indicating the concordance between genomic and transcriptomic ITH. Moreover, AML patients with low cytogenetic or molecular risk had a low ITH, and the patients with intermediate/high cytogenetic or molecular risk showed higher ITH score (Fig. 2I). Notably, we found that the percentage of blasts did not correlate with ITH, indicating that the number of leukemia cells did not contribute to the intratumoral cellular diversity (Supplementary Fig. S2I). We found that the percentage of blasts in bone marrow was not related to the overall survival (OS) of AML patients in the TCGA cohort (Fig. 2J), but the higher ITH could predict the poor OS and higher relapse rate of AML patients (Fig. 2K, L). This observation suggested that rather than the amount, the diversity of leukemia cells was associated with the outcomes of AML patients.

In order to further evaluate the additional prognostic power of our ITH-index for AML wild-type patients, TCGA samples were divided into four classes for each gene mutation, including mutated/ITH-high, mutated/ITH-low, wild-type/ITH-high, and wild-type/ITH-low. The results showed that a high level of ITH-index could predict the poor OS of TP53, DNMT3A, RUNX1, ASXL1, KRAS, and FLT3 wild-type AML patients (Supplementary Fig. S3A). Furthermore, we also found ITH-index could subdivide the outcomes of the intermediate-risk group categorized by cytogenetic or molecular alterations (Supplementary Fig. S3B). Therefore, these results suggested our ITH-index derived from single-cell transcriptomic data had an additional prognosis value, not simply mirroring the known impacts of genetic abnormalities.

### QSCs are involved in the chemoresistance and poor outcomes of AML

**Fig. 3:**
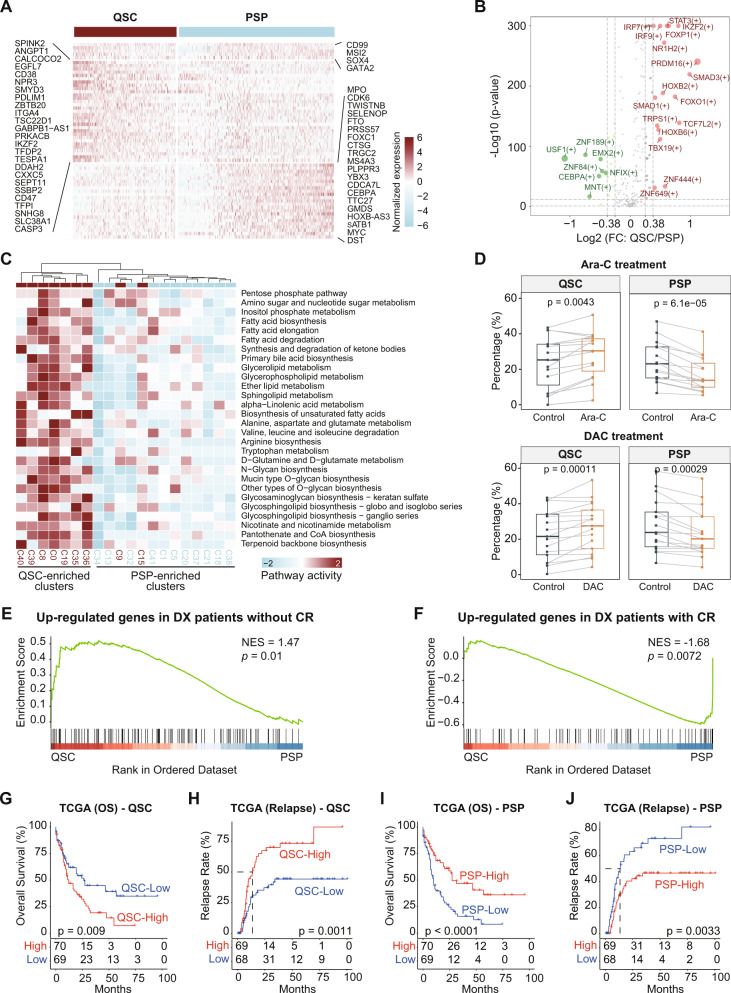
Comparing the characteristics between QSC and PSP cellular states. **A** Heatmap showing the genes expressed in the QSC and PSP cells. Both common and specific genes were shown. **B** Volcano plot showing the regulons that are specifically activated in the QSCs and PSPs. Regulons highlighted by red are significantly activated in QSCs cells, while regulons highlighted by green are significantly activated in PSPs cells. **C** Heatmap of metabolic pathways that are specifically activated in QSC-enriched clusters. **D** The proportion of the QSCs and PSPs in the control group, cytarabine (Ara-C) treated group, and decitabine (DAC) treated group in bulk transcriptomic data (GSE40442) of patient-derived AML samples. Proportions of cellular components are inferred by the deconvolution method CIBERSORTx. Paired Wilcoxon rank-sum test was used to measure the differences between groups. **E**, **F** GSEA of the differentially expressed genes between QSCs and PSPs in the gene signatures from diagnostic AML patients with/without CR. Dataset from GSE103424. **G**, **H** Kaplan–Meier analysis of OS (**G**) and relapse (**H**) of TCGA AML patients. All patients were categorized into two groups based on the median of QSCs percentage. **I**, **J** Kaplan–Meier analysis of OS (**I**) and relapse (**J**) of TCGA AML patients. All patients were categorized into two groups based on the median of PSPs percentage.

We found that AML patients with high enrichment of QSCs tended to have higher ITH, while the patients with high enrichment of PSPs tended to have lower ITH (Fig. 2M). Therefore, we compared the gene expression profile between QSCs and PSPs. Results showed that the QSCs and PSPs commonly expressed primitive markers *CD99*, *MSI2*, and *SOX4*. Besides, QSCs had significantly high expression of *EGFL7*, *IKZF2* and *CD47* (Fig. 3A and Supplementary Fig. S4A). Previous studies demonstrated that the increased expression and secretion of proangiogenic factor EGFL7 could support the growth of leukemic blasts, and a high level of *EGFL7* was associated with lower CR rates, shorter event-free, and OS in AML patients [32]. Consistently, EGFL7 contains the intronic miR-126, which could preserve LSC in a quiescent state via inhibiting PI3K/AKT signaling, promoting AML chemoresistance [33, 34]. Also, the chromatin remodeler *IKZF2* was recently reported to drive LSC self-renewal and inhibit myeloid differentiation through disrupting the CEBPD/E-driven transcriptional regulation program [35]. The “don’t eat me” signal *CD47* was proved to be an essential mechanism of immune evasion in hematological malignancies [36]. These results suggested that the QSCs highly expressed genes involved in LSC self-renewal, differentiation blockage, immune evasion, and angiogenesis. On the other hand, compared with QSCs, PSPs tended to express the genes associated with the cell cycle, such as CDK6.

To determine the association between cellular state enrichment and genomic events, we compared the chromosomal abnormality and gene mutation occurrence in patients with high-QSCs and low-QSCs percentages. RUNX1, TP53, and DNMT3A mutations were found to be enriched in the patients with a high percentage of QSCs, while all of the patients with t(15;17) had a low level of QSCs (Supplementary Fig. S4B). In contrast, patients with a high percentage of PSPs showed enrichment of t(15;17), t(18;21), and CEBPA mutation, while almost no RUNX1 mutation (Supplementary Fig. S4C). AML patients harboring mutations in TP53, RUNX1, and DNMT3A had a high risk of disease progression and poor outcome, whereas the patients with t(15;17) and t(18;21) had a relatively favorable outcome [37]. These results suggested that the patients with high-risk genomic events tended to have a higher level of QSCs, but those with low-risk genomic events tended to have a higher level of PSPs.

We further identified the transcriptional regulons that were distinctly activated in QSCs or PSPs. Results showed that more regulons were activated in QSCs than in PSPs, indicating the complicated regulation network in QSCs (Fig. 3B). The transcriptional programs regulated by STAT3, IKZF2, PRDM16, and SMAD3 were found to be significantly activated in QSCs. Notably, both the IKZF2 expression on mRNA level and the activity of its target genes were upregulated in QSCs. Also, the high expression level of *PRDM16* was reported to involve pathological progression and poor prognosis of AML, and the downregulation of *PRDM16* mRNA had an anti-leukemia effect in mice [38]. Combined with these previous studies, our results suggested that the transcriptional regulators of QSCs might be the potential therapeutic targets of RR-AML. Besides, a considerable number of metabolic pathways in QSCs were activated compared with PSPs (Fig. 3C), including pentose phosphate pathway (PPP) and arginine, fatty acid, nicotinamide metabolism pathways. In addition, by performing GSEA analysis using the differential expressed genes between QSCs and PSPs, we found that QSCs exhibited significant enrichment for some previously identified chemoresistance signatures, such as fatty acid metabolism and senescence-like signatures (Supplementary Fig. S4D).

To further demonstrate the relationship between cellular states and drug response, we used CIBERSORTx [31] to analyze the remodeling induced by chemotherapy cytarabine (Ara-C) or hypomethylating agents Decitabine (DAC) by inferring the cellular components of patient-derived AML samples based on bulk transcriptomic data (GSE40442) [39]. Compared with the control groups, the Ara-C or DAC treated groups had significantly increased QSCs and decreased PSPs proportions (Paired Wilcoxon rank-sum test, *p* value < 0.01), suggesting the enrichment of QSCs in the chemo-residual cells (Fig. 3D). Furthermore, we found that the QSCs signature was enriched in the upregulated genes of AML patients without CR after the “7 + 3” regimen, whereas the PSPs signature was enriched in the upregulated genes of patients who eventually achieved CR (Fig. 3E, F), indicating that QSCs are related to the chemoresistance of AML patients, and PSPs are more sensitive to chemotherapy. By comparing the cellular components at diagnosis and relapse in matched samples from four independent datasets, we found that the percentage of QSCs significantly increased at relapse (Supplementary Fig. S4E). As shown in Fig. 3G–J, patients with a high level of QSCs showed poor OS (log-rank test, *p* = 0.009) and high relapse rate (log-rank test, *p* = 0.0011). In contrast, patients with a high level of PSPs had favorable OS (log-rank test, *p* < 0.0001) and low relapse rate (log-rank test, *p* = 0.0033). These results were also maintained when the APL cases were excluded (Supplementary Fig. S4F, G). We validated this result in an independent cohort, and the trend was consistent with the observations in the TCGA cohort (Supplementary Fig. S4H–K). Finally, we demonstrated that the OS of QSC^High^PSP^Low^ patients was significantly poorer than QSC^Low^PSP^High^ patients (log-rank test, *p* = 0.00012; Supplementary Fig. S4L).

### PSPs obtain QSC-like transcriptional patterns after chemotherapy in refractory patients

**Fig. 4:**
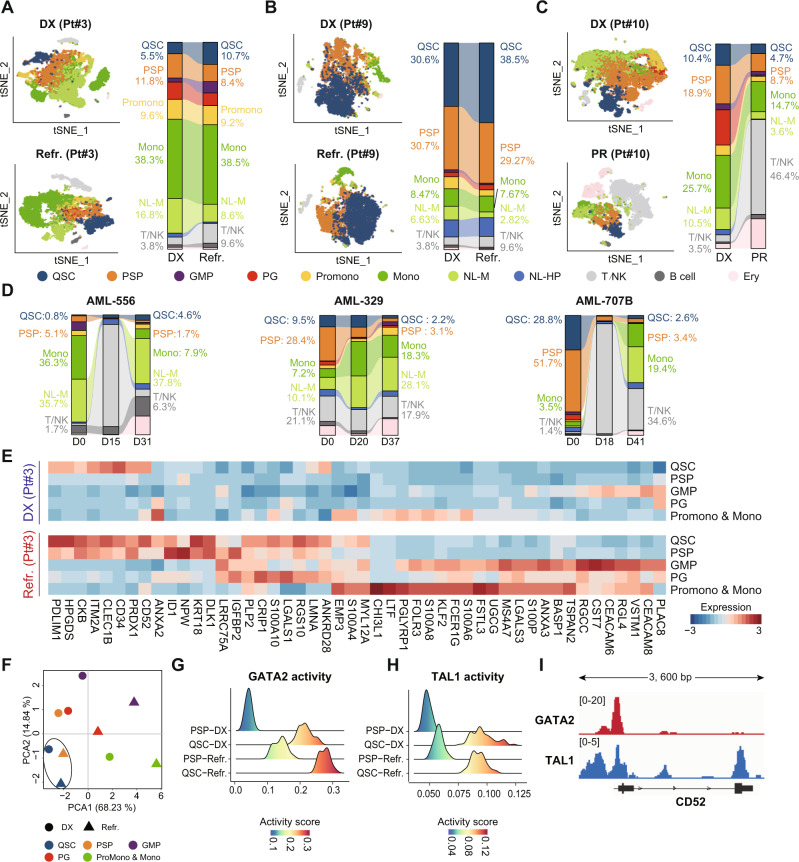
Longitudinal scRNA-seq analyses for the identification of cellular and molecular reprogramming induced by chemotherapy in AML patients. **A** Left: t-SNE visualization of scRNA-seq data of primary refractory patient Pt#3 before and after chemotherapy. Right: Sankey plot showing the dynamic changes of cellular states after chemotherapy in Pt#3. **B** Left: t-SNE visualization of scRNA-seq data of primary refractory patient Pt#9 before and after chemotherapy. Right: Sankey plot showing the dynamic changes of cellular states after chemotherapy in Pt#9. **C** Left: t-SNE visualization of scRNA-seq data of Pt#10 who achieved partial remission. Right: Sankey plot showing the dynamic changes of cellular states after chemotherapy in Pt#10. **D** Sankey plot showing the dynamic changes of cellular states during chemotherapy in samples from the study of Galen et al. **E** Heatmap for differentially expressed genes after chemotherapy in Pt#3. **F** Principal component analysis (PCA) of the cellular states in Pt#3 based on the differentially expressed genes during chemotherapy. The dot shape represents the cellular states before or after chemotherapy. **G**, **H** Ridge plots showing the activity scores of transcriptional factors GATA2 and TAL1 in QSCs and PSPs during chemotherapy in Pt#3. **I** The enrichment of GATA2 and TAL1 peaks on gene CD52 based on the ChIP‐Seq data of K562 cell line.

Next, we sought to validate the above observations by performing longitudinal scRNA-seq analysis in matched samples from two primary refractories, one partial remission (PR), and three CR AML patients during the chemotherapy. Figure 4A showed the cellular states at diagnosis and after chemotherapy in monocytic AML Pt#3, who showed chemoresistance after two courses of treatment. We found that the percentage of PSPs decreased after chemotherapy, but the percentage of QSCs increased from 5.5% to 10.7%, suggesting that the QSCs were resistant to chemotherapy, and PSPs were relatively sensitive to chemotherapy. Besides, the changes of cellular states in primary refractory patient Pt#9 also showed an increase of QSCs after chemotherapy (Fig. 4B), whereas both QSCs and PSPs decreased by half after chemotherapy in patient Pt#10 who achieved PR (Fig. 4C). To further validate the above observations, we defined the cellular states in longitudinal scRNA-seq samples from Galen et al.’s study [19] by a label-transform algorithm (Fig. 4D). Although having achieved clinical CR, AML-556 showed a gradual increase of QSCs percentage along with the chemotherapy procedure, from 0.8% at diagnosis to 2.6% at D15 and 4.6% at D31, further indicating the potential role of QSCs in chemoresistance. Besides, in spite that the QSCs in CR patient AML-329 decreased from 9.5% (D0) to 2.2% (D37), they remained almost unchanged at the D20 after 7 + 3 induction chemotherapy. In contrast, the PSPs were significantly reduced by chemotherapy at D20, suggesting that the QSCs had a late response compared to PSPs. Collectively, our observations showed that QSCs were obstinate or late-responsive to chemotherapy, suggesting the involvement of QSCs in AML chemoresistance.

Some previous studies reported a decrease in LSC signatures in chemo-residual AML cells [13, 14, 40]. Consistently, our longitudinal single-cell analysis of AML-329 and AML-707B suggested that the stemness of residual AML cells significantly decreased at the cytoreduction period (about D20 after chemotherapy) compared to the pre-treatment, and then recovered at about D40 (Supplementary Fig. S5A). But in AML-556, both QSCs and PSPs showed significantly increased stemness at D15 and D31 compared to pre-treatment, possibly due to the differential response timing among individuals. In contrast, signatures of inflammatory response and chemokine signaling pathway significantly increased at the initial cytoreduction period and decreased afterward, suggesting that these cellular inflammatory responses were transient (Supplementary Fig. S5B). Therefore, these results suggested that cellular states were dynamically changed during chemotherapy. The residual AML cells experienced an inflammatory response under chemical stress, during which the stemness of these residual cells was decreased. These inflammatory responses would attenuate and stemness would recover, but the response timing may be differential among AML individuals.

In order to further figure out the underlying cellular and molecular mechanism of chemoresistance, we analyzed the transcriptional reprogramming induced by chemotherapy in refractory patients by identifying the differential expressed genes of chemo-residual cells compared to cells before treatment (Fig. 4E and Supplementary Fig. S6A). Notably, we found that the residual PSPs obtained transcriptional features similar to QSCs after treatment. For example, the upregulation of the QSC-signature genes *LGALS1*, *CD52*, and *PDLIM1* were observed in PSPs after chemotherapy. The principal component analysis (PCA) also showed that the gene expressions of PSPs were more similar to QSCs after chemotherapy than the initial diagnosis status in primary refractory patients (Fig. 4F). We next analyzed the dynamic changes in transcriptional regulations during chemotherapy treatment and identified the potential factors involved in the reprogramming of PSPs. Results showed that PSPs had a relatively low level of GATA2 activity and expression at diagnosis, while these levels in QSCs were significantly higher (Fig. 4G and Supplementary Fig. S6B), largely due to the crucial role of GATA2 in stemness maintenance of LSCs [41, 42]. Moreover, both the activity and expression level of GATA2 increased significantly in PSPs after chemotherapy. Besides, we also found that PSPs obtained the activated regulon controlled by factor TAL1 (Fig. 4H and Supplementary Fig. S6C), which was required to maintain the quiescent state of LSCs [43]. Overall, these results showed the acquisition of a QSC-like transcriptional program in chemotherapy-induced PSPs, indicating that cellular state shifting may be an important mechanism for AML chemoresistance.

**Fig. 5:**
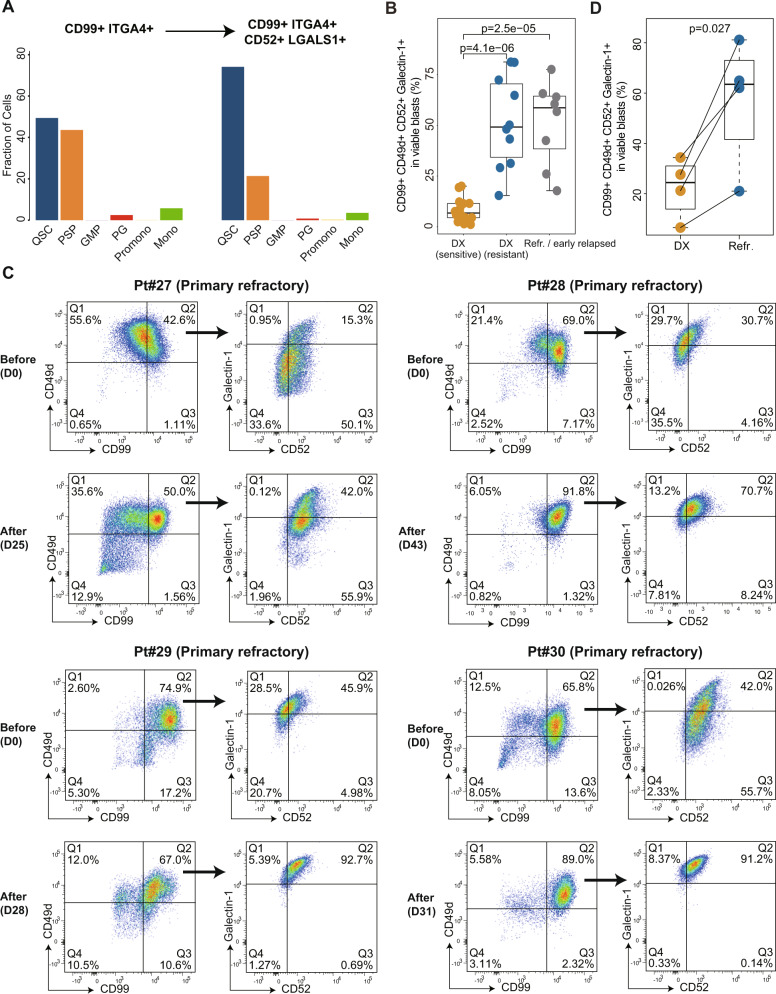
Validation of the enrichment of QSCs in refractory and early-relapsed AML patients by flow cytometry based on the markers identified by single-cell data. **A** Bar plots showing the percentage of each leukemia-like cellular state in CD99^+^ITGA4^+^ and CD99^+^ITGA4^+^CD52^+^LGALS1^+^ cells of single-cell transcriptomic data in this study. **B** Comparison of the percentage of CD99^+^CD49d^+^CD52^+^Galectin-1^+^ (QSCs) cells in viable CD45^dim^SSC^low^ blasts among diagnosis (DX) sensitive patients, diagnosis (DX) resistant patients, and refractory/early-relapsed AML patients by flow cytometry. **C** Flow cytometric analysis of the percentage of QSCs in matched bone marrow samples from primary refractory AML patients (Pt#27, Pt#28, Pt#29, and Pt#30) before and after chemotherapy. **D** Comparison of the percentage of QSCs in viable CD45^dim^SSC^low^ blasts at diagnosis and refractory bone marrow samples of Pt27, Pt#28, Pt#29, and Pt#30.

Notably, we found that the currently targetable gene *CD52* was transcriptionally regulated by GATA2 and TAL1 in AML cells (Fig. 4I), and the expression of *CD52* was positively correlated with the activity of these regulators in single cells (Supplementary Fig. S6D, E). We further validated the enrichment of QSCs in chemoresistant patients by flow cytometry based on the markers identified by our single-cell data. Specifically, we identified the cell surface markers CD99 and ITGA4 (CD49d) that were specifically expressed on both QSCs and PSPs to recognize these two subpopulations first, and then used CD52 and LGALS1 (Galectin-1) to further distinguish QSCs from PSPs (Fig. 5A). We used flow cytometry to test the bone marrow samples from 24 diagnostic AML patients (including 14 patients that achieved CR and ten patients resistant to chemotherapy) and eight samples from refractory/early-relapsed (<6 months) AML patients. Results showed that CD99^+^CD49d^+^CD52^+^Galectin-1^+^ (QSCs) cells had significantly higher levels in viable CD45^dim^SSC^low^ blasts of diagnosis resistant and refractory/early-relapsed patients than diagnostic patients who eventually achieved CR after chemotherapy (Fig. 5B). This observation validated that QSCs contributed to the chemoresistance of primary refractory and early-relapsed AML patients. We further performed flow cytometric analysis for matched bone marrow samples from four primary refractory patients during chemotherapy (Fig. 5C). Results showed that the percentage of CD99^+^CD49d^+^CD52^+^Galectin-1^+^ (QSCs) cells remarkably increased in chemo-residual resistant cells, compared with the diagnostic samples (paired *t*-test, *p* = 0.027; Fig. 5D). These observations suggested that the preexisting CD99^+^CD49d^+^CD52^+^Galectin-1^+^ (QSCs) cells at diagnosis were chemoresistant, and these cells were further enriched in the persisting residual AML cells.

### Interaction of CD52-SIGLEC10 between QSCs and monocytes may contribute to the poor outcome of AML

**Fig. 6:**
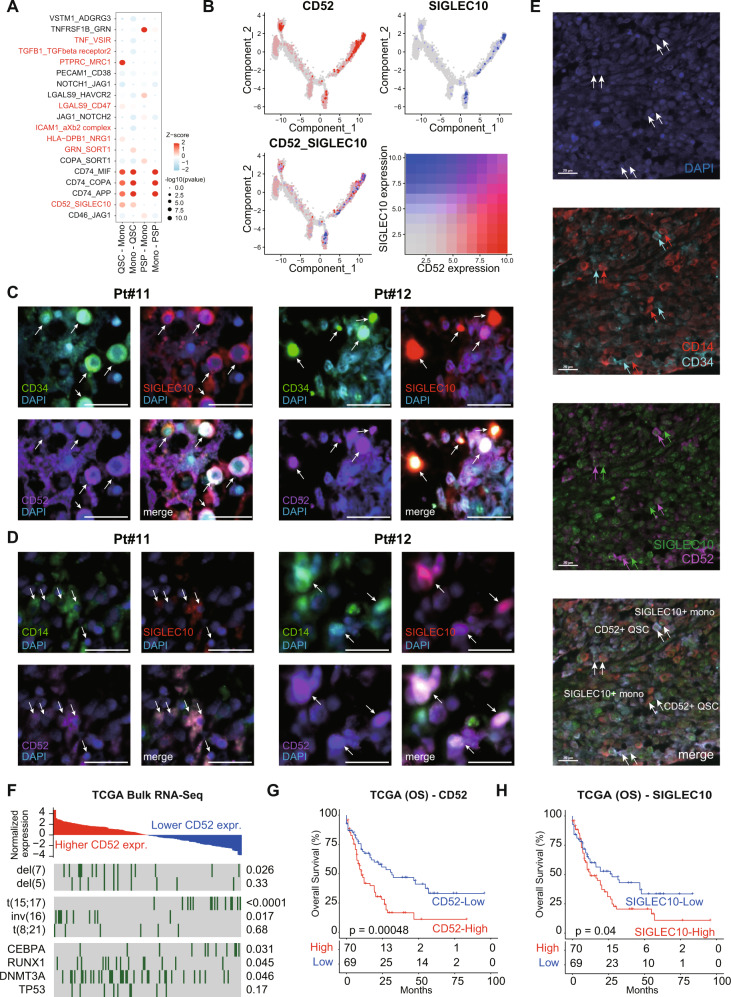
Cell–cell communications between QSCs and monocytes by CD52-SIGLEC10 interaction. **A** Bubble chart showing the ligand-receptor interactions between QSCs/PSPs and monocytes in RR-AML patients. **B** Expression of CD52 (gray to red) and SIGLEC10 (gray to blue) on the pseudotime map of leukemia-like cells. **C** Bone marrow samples from AML patient Pt#11 and Pt#12 stained by anti-CD34 (green), anti-SIGLEC10 (red), anti-CD52 (purple) antibodies. DAPI (blue) is used for staining the live cells. Arrows depict the CD34^+^CD52^+^SIGLEC10^+^ QSCs. Scale bar, 20 μm. **D** Bone marrow samples from AML patient Pt#11 and Pt#12 stained by anti-CD14 (green), anti-SIGLEC10 (red), anti-CD52 (purple) antibodies. DAPI (blue) is used for staining the live cells. Arrows depict the CD14^+^CD52^+^SIGLEC10^+^ monocytes. Scale bar, 20 μm. **E** Multiplex IHC shows the interactions between CD34^+^CD52^+^ QSCs and CD14^+^SIGLEC10^+^ monocytes. **F** Relationship between CD52 expression levels and genomic events in TCGA AML cohort. Fisher exact test was used to measure the differences between groups. **G**, **H** Kaplan–Meier analysis of OS of TCGA AML patients. All patients were categorized into two groups based on the median level of CD52 (**G**) or SIGLEC10 (**H**) expression.

Benefiting from the advantages of scRNA-seq technology, we could perform a high-resolution dissection of the communications among various cell types in the microenvironment of RR-AML bone marrow. The differentially activated interaction pathways of QSCs and PSPs were identified based on the expression of ligands and receptors on single cells. Notably, results showed that CD52 and its receptor SIGLEC10 interaction was significantly enriched in the communications between QSCs and monocytes (Fig. 6A). By tracing the expression distribution along the single-cell trajectory, *CD52* and *SIGLEC10* showed specific co-expression in QSCs and mono cells (Fig. 6B). However, PSPs had no such co-expression. Multiplex immunofluorescence analysis further validated the existence of *CD52*^+^*SIGLEC10*^+^ QSCs and monocytes in AML patients Pt#11 and Pt#12 (Fig. 6C, D). Furthermore, multiplex staining of CD34, CD14, CD52 and SIGLEC10 visualized the interaction between CD34^+^CD52^+^ QSCs and CD14^+^SIGLEC10^+^ monocytes (Fig. 6E). CD52 was reported as a small glycoprotein that could suppress T-cell activation [44] and inflammatory cytokines produced by monocytes [45]. By analyzing the *CD52* expression of the TCGA cohort (Fig. 6F), we found that AML patients with del(7), inv(16), RUNX1 and DNMT3A mutations showed significantly higher *CD52* expression, implying that the patients harbored these genetic alterations may be suitable for the treatment of CD52 targeted antibody alemtuzumab. These observations were further validated in the BeatAML cohort (Supplementary Fig. S7A). Consistently, several previous studies also reported the effect of targeting CD52 in a subset of AML patients [46, 47].

We observed that QSCs showed a significantly increased *CD52* expression after chemotherapy in the primary refractory patients (Supplementary Fig. S7B). Moreover, AML patients with higher *CD52* expression had a significantly worse OS in the TCGA dataset (log-rank *p* = 0.00048; Fig. 6G). Notably, *SIGLEC10*, the receptor of *CD52*, was also associated with the adverse prognosis of AML (log-rank *p* = 0.04; Fig. 6H), further suggesting the important role of CD52-triggered signaling in AML progression.

### LGALS1 inhibitor is an effective way to target QSCs and enhance the chemotherapy for refractory AML patients

**Fig. 7:**
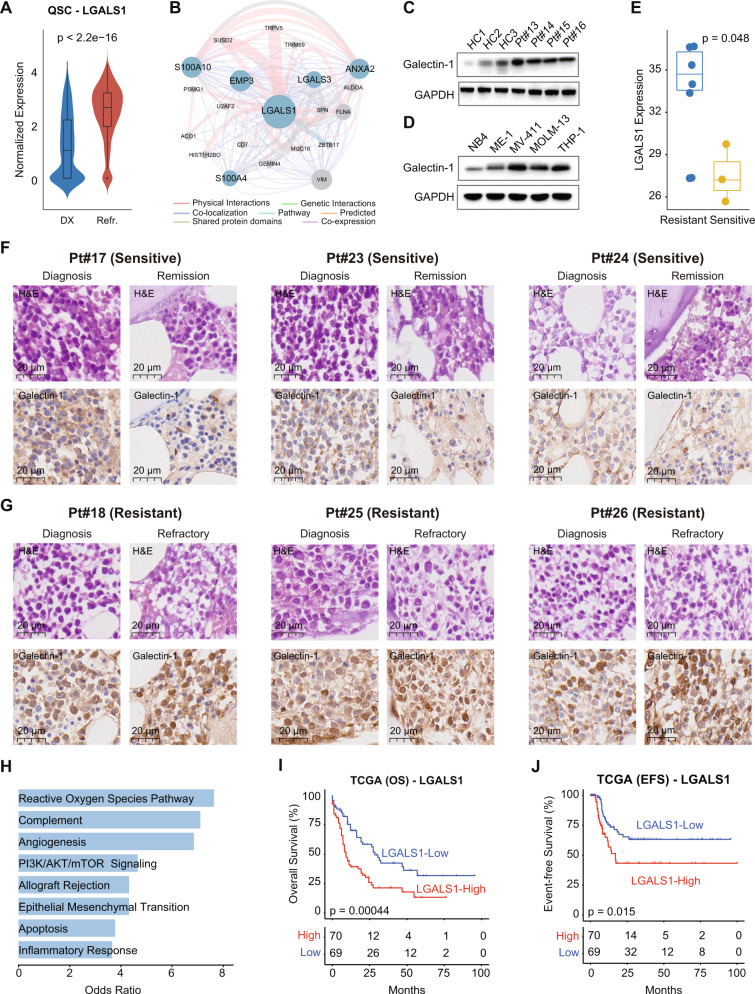
The role of LGALS1 in AML chemoresistance. **A** The expression level of LGALS1 in the QSCs of Pt#3 before and after chemotherapy. Wilcoxon rank-sum test was used to measure the differences between groups. **B** The functional module involved by LGALS1 based on GENEMANIA. Upregulated genes in the refractory sample were highlighted by the blue node. **C** Western blotting showing the protein level of Galectin-1 in HC individuals and AML patients. **D** Western blotting showing the protein level of Galectin-1 in various AML cell lines. **E** Boxplot showing the difference of LGALS1 expression in resistant and sensitive AML cell lines. Wilcoxon rank-sum test was used to measure the differences between groups. **F**, **G** IHC staining for Galectin-1 in chemo-sensitive (**F**) and chemoresistant AML patients (**G**). Both diagnosis and remission/refractory samples were shown, with scale bars indicated for all sections. **H** Barplot showing the functional hallmarks that were enriched by the genes co-expressed with LGALS1. **I**, **J** Kaplan–Meier analysis of OS (**I**) and EFS (**J**) of TCGA AML patients. All patients were categorized into two groups based on the median of the LGALS1 expression.

As one of the most upregulated genes in QSCs cells in RR-AML (Fig. 2E and Supplementary Fig. S8), LGALS1 also showed a significantly increased expression in chemo-residual QSCs in refractory AML patients (Wilcoxon rank-sum test, *p* < 2.2e–16; Fig. 7A). By performing network analysis based on physical interactions and co-expression, we demonstrated that the upregulated genes LGALS1, S100A10, EMP3, and S100A4 in the residual leukemia-like cells were involved in a functional module with LGALS1 as a hub gene (Fig. 7B). The protein expression level of LGALS1 (Galectin-1), was higher in the peripheral blood mononuclear cells (PBMC) of AML patients than in healthy donors (Fig. 7C). AML cell lines MV-411 and THP-1 expressed a relatively high level of Galectin-1 (Fig. 7D). In addition, using the RNA-seq data from Williams et al. [48], we found that the *LGALS1* expression was remarkably higher in daunorubicin-resistant leukemia cell lines than in the sensitive cells (Wilcoxon rank-sum test, *p* = 0.048; Fig. 7E), indicating the potential role of *LGALS1* in drug resistance. We further performed IHC experiments in bone marrow samples from AML patients with different chemo-sensitivity to validate the involvement of Galectin-1 in AML chemoresistance. The clinical and genetic characteristics of these patients were shown in Supplementary Table S3. Results showed that the level of Galectin-1 was higher in chemoresistant patients than in sensitive patients at diagnosis. Moreover, the Galectin-1 expression was even higher in the chemo-residual cells in resistant patients (Fig. 7F, G), consistent with our transcriptional observations at the single-cell level. Furthermore, we found that the co-expression module involved by *LGALS1* enriched in the reactive oxygen species pathway and PI3K/AKT/mTOR signaling (Fig. 7H), implying that *LGALS1* may mediate the chemoresistance through the activation of these biological pathways. Survival analysis suggested that the higher expression of LGALS1 was associated with the poor OS (log-rank *p* = 0.00044; Fig. 7I) and EFS (log-rank *p* = 0.015; Fig. 7J) of AML patients in the TCGA cohort.

**Fig. 8:**
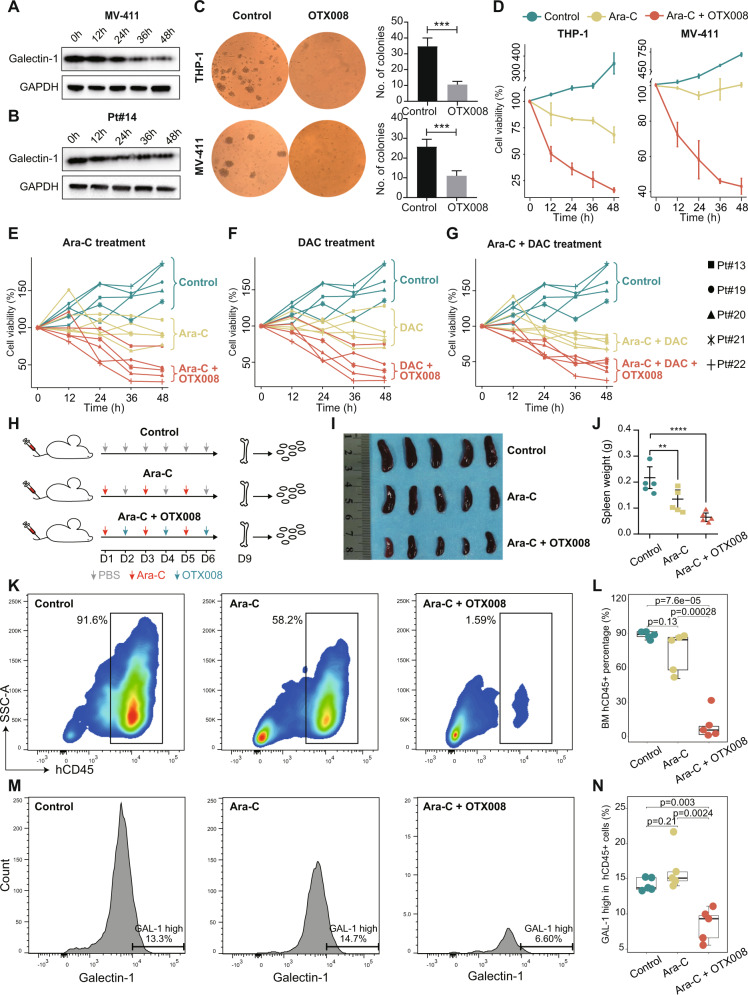
The chemosensitizing efficacy of LGALS1 inhibitor OTX008 in AML cells and xenograft model. **A**, **B** Western blot analysis of Galectin-1 expression in AML cell line MV-411 (**A**) and patient Pt#14 (**B**) after treatment with 30 µM OTX008 for different time points (0, 12, 24, 36, and 48 h). **C** Colony formation assays of AML cell line THP-1 and MV-411 with or without OTX008 treatment. Quantification of the number of colonies was shown on the right, ***P < 0.001. **D** CCK-8 assay showing the cell viability of THP-1 and MV-411 cells incubated with DMSO, Cytarabine (200 nm), or Cytarabine (200 nm) + OTX008 (30 µM) for different time points (0, 12, 24, 36, and 48 h). **E**–**G** CCK-8 assay showing the cell viability of PBMC from five refractory AML patients during treatment of different strategies for different time points (0, 12, 24, 36, and 48 h). **H** Scheme showing the process of engrafting human primary AML cells in NCG mice and drug treatment workflow. **I** The spleen images from mice of different treatment groups. **J** Spleen weights of NCGs of different treatment groups. *P* values were calculated by two-tailed *t*-test. **K** Representative flow cytometric analysis of the human leukemic burden in bone marrows from NCG mice of different treatment groups. **L** Quantification of human leukemic burden (hCD45^+^ cells relative to the total cells) in the bone marrow of NCG mice post-treatment of different groups. **M** Representative flow cytometric analysis of cells with high Galectin-1 expression in hCD45^+^ cells from bone marrows of different treatment groups. **N** Percentage of cells with high Galectin-1 expression in hCD45^+^ cells from bone marrows of different treatment groups.

We next investigated the efficacy of Galectin-1 inhibitor OTX008 on the primary patient-derived AML cells and cell lines. The level of Galectin-1 decreased along with the time of OTX008 treatment in cell line MV-411 and Pt#14 (Fig. 8A, B). The colony formation ability was evaluated before and after OTX008 treatment. As shown in Fig. 8C, the number of colonies significantly decreased after OTX008 treatment in THP-1 and MV-411 cell lines, demonstrating the efficacy of the Galectin-1 inhibitor in inhibiting the self-renewal of AML cells. We evaluated the chemosensitizing efficacy of LGALS1 inhibitor in AML cells with different clinical and genetic characteristics (Supplementary Tables S4 and S5), including both cell lines and patient-derived primary cells. Results showed that combined treatment of LGALS1 inhibitor OTX008 and Ara-C significantly reduce the cell viability of AML cell line THP-1, MV-411, Kasumi-1, ME-1, HL-60, KG-1 and MOLM-13, compared with Ara-C alone (Fig. 8D and Supplementary Fig. S9). Besides, flow cytometric analysis also showed a significant increase of cell apoptosis in the combined treatment group (Supplementary Fig. S10). Furthermore, we treated the mononuclear cells from bone marrow samples of five refractory AML patients to further validate the chemotherapy enhancement effect. Results showed that the combination of OTX008 with Ara-C/DAC had a significant effect on inhibiting the cell viability of AML than the single agent of Ara-C or DAC (Fig. 8E–G). In summary, our in vitro analysis indicated that the Galectin-1 inhibitor could significantly sensitize both primary AML cells and cell lines to chemotherapy.

We further validate the inhibiting of leukemia burden and QSCs by combined treatment of Ara-C and OTX008 in xenograft models of human AML. Human Primary AML cells were injected intravenously into NCG mice to construct AML xenograft models followed by vehicle (PBS), Ara-C, or combined treatment (see Methods, Fig. 8H). Results showed that combined treatment of Ara-C and OTX008 significantly decreased the leukemia burden in the spleen, compared to control and Ara-C alone groups (Fig. 8I, J). Flow cytometric analysis of mice bone marrow samples showed that human leukemia blasts (hCD45^+^) reduced significantly in the combined treatment group (Fig. 8K, L). In addition, compared to the other groups, human xenografts from the combined treatment group had the lowest level of Galectin-1^high^ cells (Fig. 8M, N), as well as the lowest percentage of CD99^+^CD49d^+^CD52^+^Galectin-1^+^ cells (Supplementary Fig. S11), indicating the inhibitory effect on QSCs.

## Discussion

This study aims to reveal the biological features of AML chemoresistance and identify the potential strategies for chemotherapy enhancement. Our work is distinctive from the previous studies in several crucial ways. Firstly, we dissected the heterogeneity of LSCs in primary refractory and short-term relapsed AML patients at the single-cell level and identified subpopulations with different chemotherapy sensitivity. Secondly, our study showed that chemotherapy-induced cellular plasticity contributed to the resistance of refractory AML patients by performing longitudinal scRNA-seq analyses. Thirdly, we observed the intercellular interactions between QSCs and monocytes at high resolution and validated by multiplex IF staining of bone marrow samples from AML patients. Finally, we demonstrated the unappreciated role of LGALS1 in AML chemoresistance by single-cell analysis, flow cytometry and IHC experiments in human bone marrow samples, as well as indicated that QSCs could be chemo-sensitized by LGALS1 inhibitor in vitro and in vivo. Collectively, the single-cell transcriptional characterization in this study suggests that the chemotherapy is selective for AML subpopulations, and the analysis of transcriptional plasticity could provide potential molecules that could be targeted for RR-AML. To the best of our knowledge, previous scRNA-seq studies of AML mainly focused on diagnostic or relatively late-relapsed AML patients [9, 10, 19, 21, 49], the present study is the largest single-cell dataset for primary refractory and short-term relapsed AML patients thus far, facilitating to identify the critical factors in AML chemoresistance.

With different genetic characteristics or FAB subtypes, AML is a kind of disease with highly inter-tumoral heterogeneity based on the WHO classification [50]. However, cytarabine-based chemotherapy is the most commonly used treatment strategy in a majority of AML subtypes until now [51, 52]. Revealing the common biological features of chemoresistance for AML patients with different subtypes is important to develop potential strategies for chemotherapy enhancement, especially for patients without known driver mutations and available targeted therapies. Based on this consideration and consistent with the previous studies which focused on AML chemoresistance [13, 14, 40], we included RR-AML patients with different genetic/FAB subtypes but received the same treatment regimens in this study. To confirm the generalization and reliability of conclusions, the findings derived from the single-cell analysis were further validated in multiple independent public datasets and experiments performed on AML samples with different subtypes, including ten independent datasets, flow cytometry for 40 samples, IHC for 12 paired samples, combined treatment for 7 cell lines and 5 primary samples of RR-AML. These pieces of evidence consistently proved that the QSCs identified at the single-cell resolutions played a critical role in AML chemoresistance and targeting QSCs could sensitize chemotherapy in AML cells with different subtypes.

In line with some previous studies [7–9], we observed that the stem-like leukemia cells in the absence of proliferation were resistant to chemotherapy and related to a high risk of relapse and poor survival. However, studies from Farge et al. [13] and Duy et al. [14] characterized the AML cellular changes during chemotherapy, demonstrating that AML chemoresistance is driven by fatty acid metabolism and senescence-like status, respectively, rather than by quiescent LSCs. These controversial conclusions may partially arise from the high heterogeneity of the LSC group which could not be fully deconstructed by previously used bulk sequencing. Notably, the QSCs in our single-cell data also exhibit significant enrichment for fatty acid metabolism and senescence-like signatures compared with PSPs, consistent with the characterization of chemoresistant cells in these two studies. Thus, the quiescent LSC status and fatty acid metabolism/senescence are not mutually exclusive. Besides, the chemotherapy-induced dynamic cellular plasticity may also lead to controversial conclusions regarding the characteristics of residual AML cells. The cellular and molecular features dynamically change during chemotherapy and experience a procedure of “pre-treatment → initial cytoreduction → regeneration → relapse” [40]. AML cells respond under biochemical stress conditions and have increased pro-inflammatory [7, 13, 14] and decreased LSC signatures [14, 40] during the initial cytoreduction and regeneration period. Studies based on the AML in vivo model also demonstrate that these immediate cellular responses are transient and would attenuate within about 1 week after chemotherapy, and then the expression of LSC-related genes would recover [14]. Collectively, the nature of the high heterogeneity of the LSC group, as well as the chemotherapy-induced dynamic plasticity leads to controversial conclusions in the field. The longitudinal single-cell analysis of AML patients would provide insight into the dynamic cellular and molecular reprogramming of various LSC subpopulations during chemotherapy.

Notably, by tracing the dynamic transcriptional changes induced by chemotherapy in AML patients, we showed that PSPs were reprogrammed to get a similar expression pattern to QSCs. This result coincided with a recent study that elucidated chemotherapy could induce a cell-cycle arrest and growth pause in leukemia [14]. These results suggest that AML cells may experience cellular state transition to avoid cell death induced by chemotherapy and stand by to reconstitute leukemia. Combined with the results from the previous study [14], we speculated that this cellular reprogramming induced by chemotherapy might be dose-dependent. Under the treatment dose that cannot kill them, PSPs would obtain the QSC-like transcriptional program and get more resistant. Although high-dose chemotherapy could increase early remission and survival in several clinical trials [53, 54], the adverse effects are non-negligible, especially in older patients. Therefore, the addition of treatment that targets chemotherapy-induced QSCs might improve the efficacy of low-dose or standard-dose chemotherapy.

Our study indicated that the currently targetable genes CD52 and LGALS1 were specifically expressed in QSCs, and showed increased expression in chemo-residual QSCs and PSPs of RR-AML patients. The interaction of CD52-SIGLEC10 between QSCs and monocytes may contribute to the immune evading and poor outcome of AML. The roles of LGALS1 are well studied in some hematologic malignancies, such as chronic lymphocytic leukemia (CLL) and lymphoma [55, 56], but the understanding of LGALS1 in AML pathology and progression is currently limited. Ruvolo et al. reported that mice bearing the OCI-AML3 cells with LGALS1 shRNA survived longer than mice with control OCI-AML3 cells, suggesting that LGALS1 suppression could prolong the survival of in vivo treatment-naïve models [57]. However, the evaluation of LGALS1 expression in primary cells or bone marrow samples from AML patients has yet to be performed. Besides, the association between LGALS1 expression and chemotherapy resistance of AML was unknown. Our study revealed the unappreciated role of LGALS1 in chemoresistant AML patients and demonstrated the chemosensitizing efficacy of inhibiting LGALS1 in patient-derived primary AML cells, cell lines, and AML xenograft models. Therefore, our study suggested that therapeutic strategies targeting CD52 and LGALS1 could help eliminate the chemoresistant QSCs.

In summary, by performing scRNA-seq analysis, this study sheds new light on the cellular and molecular reprogramming induced by chemotherapy in primary refractory and short-term relapsed AML patients and identified the cell subpopulation and molecules involved in the drug resistance and poor outcome of AML. Our results will facilitate a better understanding of the AML chemoresistance mechanism and the development of novel therapeutic strategies for RR-AML patients.

## Methods

### Human patient samples

Bone marrow aspirates from AML patients were obtained from the First Affiliated Hospital of Nanjing Medical University (Supplementary Table S1). This study was reviewed and approved by the Institutional Review Boards of Nanjing Medical University. Informed consent was obtained from each individual.

### Human cell lines and culture conditions

NB4, Kasumi-1, ME-1, MV-411, THP-1, KG-1, HL-60, and MOLM-13 leukemia cell lines were obtained from the American Type Culture Collection (ATCC). All cell lines were cultured in RPMI-1640 medium supplemented with 10% FBS, 4 mM L-glutamine, 100 units/ml penicillin, and 100 µg/ml streptomycin in a humidified atmosphere of 95% air and 5% CO_2_ at 37 °C.

### Human bone marrow single-cell suspensions preparation

Fresh human bone marrow samples of AML patients were processed using density gradient centrifugation at 500 g for 10 min at 4 °C to isolate mononuclear cells. Cell pellets were resuspended in 5 ml RPMI-1640 with 5% FBS and 2-Mercaptoethanol, and filtered using a 40 mm nylon mesh (Thermo Fisher Scientific) with residual cell clumps discarded. We used DAPI (Sigma-Aldrich) for staining to identify live cells, and cell number was determined using a Countess II Automated Cell Counter whenever possible.

### Library construction for scRNA-seq

In total, the cell viability of all samples is above 90%, 10,000 cells per sample were loaded into a Chromium Single-Cell 3′ Chip Kit v2 and processed using a Chromium single cell 3′ Reagent Kits (v2 and v3) (10× Genomics) according to the manufacturer’s instructions. Libraries were constructed using the Single 3′ Library and Gel Bead Kit v2 (PN-120237) and Chromium i7 Multiplex Kit v2 (PN-120236).

### Cell viability assay

Cell viability assays were performed with CCK-8 reagent (1:10, Dojindo, Kumamoto, Japan). According to the manufacturer’s protocol, cells (2 × 10^6^/ml) were seeded in 96-well plates and incubated with the indicated concentration of drugs. The absorbance was measured at 450 nm by spectrophotometry.

### Immunoblotting

Cells were lysed in RIPA Lysis and Extraction Buffer (25 mM Tris-HCl pH 7.6, 150 mM NaCl, 1% P-40, 1% sodium deoxycholate, 0.1% SDS) with PMSF Protease Inhibitor (Thermo Fisher Scientific) added. Ten micrograms of purified proteins were separated by SDS-polyacrylamide gel electrophoresis (SDS-PAGE) on a 15% gradient gel (BioRad) and transferred onto NC membranes (EMD Millipore). Membranes were incubated overnight at 4 °C with the following primary antibodies: rabbit anti-Galectin-1 (Abcam, ab138513, 1:1000), mouse anti-GAPDH (Abcam, ab9484, 1:5000). Anti-mouse or anti-rabbit horseradish peroxidase-conjugated antibodies (Cell Signaling Technology) were used as secondary antibodies.

### Colony formation assay

Cells were mixed with an equal volume of methylcellulose colony assay medium (MethoCult™ GF M3434, Stem Cell Technologies) and then plated in 12-well plates and incubated for 2 weeks. Colonies were calculated with a diameter of more than 0.1 mm of incubation at 37 °C in 5% CO_2_.

### Immunohistochemistry (IHC) and immunofluorescence (IF)

Formalin-fixed bone marrow tissues from AML patients were processed through the routine IHC pipeline and stained for a rabbit anti-Galectin-1 (Abcam, ab138513). Multiplex IHC staining was performed with Akoya OpalTM seven-color fluorescent platform. Formalin-fixed, paraffin-embedded tissues were cut in 4-μm thick section and stained by Opal Polaris 7 Color Automation IHC Detection Kit (Akoya Biosciences, Menlo Park, CA) for simultaneous detection and quantitation of CD34 (GenTex, GTX28158), CD14 (Novusbio, NB100-2807), CD52 (Santa Cruz Biotechnology, sc-51560), SIGLEC10 (Sigma-Aldrich, HPA027093) and DAPI (Sigma-Aldrich). The slides were observed and imaged by Vectra Polaris automated quantitative pathology imaging system. The images were spectrally unmixed by Akoya phenoptics inForm software (inform 2.4.8).

### Flow cytometric analysis

Suspended single cell was prepared from BM samples of AML patients. Red cells were lysed with 1× RBC Lysis Buffer (eBioscience, San Diego, CA, USA) before staining. Cells were incubated with antibodies in Facs buffer (2% FBS in PBS) on ice for 20 min at dark. The following human-specific monoclonal antibodies were used: CD45-Pacific Blue (BioLegend, cat. no. 304029), CD99-FITC (BioLegend, cat. no. 371303), CD49d-PE (BioLegend, cat. no. 304303), CD52-Percp-Cy5.5 (BioLegend, cat. no. 316009). For intracellular staining, surface-marked cells were fixed for 20 min and then permeabilized using an IntraStain Kit (Dako, DK) according to the manufacturer’s instructions after washing with 1× PBS (HyClone, USA) and centrifugation at 400×g for 5 min. Subsequently, the cells were stained with anti-Galectin-1 (Abcam, cat. no. ab138513) for 20 min and then washed once. Stained cells were analyzed on FACSAria (BD Biosciences, San Jose, CA, USA).

### In vivo xenograft study

Animal experiments were conducted according to protocols approved by the Institutional Animal Care and Use Committee of Nanjing Medical University. NCG (NOD/ShiLtJGpt-Prkdcem26Cd52Il2rgem26Cd22/Gpt) mice were purchased from the Nanjing Biomedical Research Institute of Nanjing University (NBRI). Briefly, NCG mice (6–8-week-old) were injected intravenously via tail vein with 2 × 10^6^ primary AML cells resuspended in 100 μL phosphate-buffered saline after being sublethally treated with busulfan (30 mg/kg/day) for 24 h. The mice were tail bled weekly to monitor the tumor burden of human cell engraftment by flow cytometry. For drug treatment, all mice were randomly divided into three groups of five mice each after 4 weeks of AML cell transplantation. Mice in the control group were treated with PBS daily for 6 days; mice in the chemotherapy group were intraperitoneally treated with Ara-C using a physiologically relevant dose and schedule (60 mg/kg/day × 6 days); mice in the co-treatment group were injected intraperitoneally with Ara-C (60 mg/kg/day) at the 1, 3, 5 days and with OTX008 (5 mg/kg/day) at the 2, 4, 6 days. Mice were sacrificed on the 9th day, bone marrow from femurs was harvested, crushed with mortar and pestle, and red blood cells lysed using ammonium chloride buffer after filtering. Cells retrieved from bone marrows were stained with mCD45-PeCy7 and hCD45-BV421 and analyzed by flow cytometry to detect the human graft (hCD45^+^mCD45^−^ population). In addition, percentages of CD99^+^CD49d^+^CD52^+^Galectin-1^+^ (QSCs) cells in human AML cells (hCD45^+^) were measured by flow cytometry to evaluate the impact of different treatment groups.

### Computational and statistical analyses

CellRanger was used to perform barcode processing and generate gene count profiles. Data integration, unsupervised clustering, visualization, and differential expression analysis were performed using Seurat [27]. Cell-type annotation was based on the expression of marker genes and singleR [58]. We used Monocle2 [59, 60] to characterize the differentiation trajectory and identify the cellular states of the leukemia-like cells. Transcriptional regulon analysis was performed utilizing pySCENIC [61, 62]. Single-cell metabolic pathway analysis was conducted by the method from Xiao et al. [63]. We evaluated the proliferation score for every single cell by using the method described by the previous study [19]. The stemness score was calculated using CytoTRACE [64]. LSC signature scores were calculated by utilizing single-sample gene sets enrichment analysis (ssGSEA). The bulk RNA-seq data and clinical information of AML patients were downloaded from TCGA, GEO, and BeatAML databases (Supplementary Table S6). We estimated the cell type abundances of bulk transcriptomic data using CIBERSORTx [31] with single-cell data in this study as a reference. Shannon entropy was used to calculate the ITH of each patient. Kaplan–Meier survival analysis was performed by the R package survminer. The log-rank test was used to evaluate the survival differences between groups. The detailed computational parameters were described in Supplementary Methods.

## Supplementary information

Supplementary Figures

Supplementary Tables

Supplementary Methods

## Data Availability

The raw sequencing data supporting this study have been deposited in the Genome Sequence Archive at National Genomics Data Center, China National Center for Bioinformation/Beijing Institute of Genomics, Chinese Academy of Sciences (https://ngdc.cncb.ac.cn/gsa-human, accession no. HRA001240). The processed count matrices have been deposited in the OMIX (https://ngdc.cncb.ac.cn/omix, accession no. OMIX002180). Custom codes and cell annotation files are accessible at GitHub: https://github.com/woolingxiang/cellular_states_of_RR-AML.
