# Supplementary material for: Single-cell analysis reveals the chemotherapy-induced cellular reprogramming and novel therapeutic targets in relapsed/refractory acute myeloid leukemia
Source: Leukemia. 2022 Dec 21;37(2):308–25. doi: 10.1038/s41375-022-01789-6 (PMC9898038; doi:10.1038/s41375-022-01789-6)
Supplement: Supplementary file 1 — Supplementary Figures [file 41375_2022_1789_MOESM1_ESM.pdf]

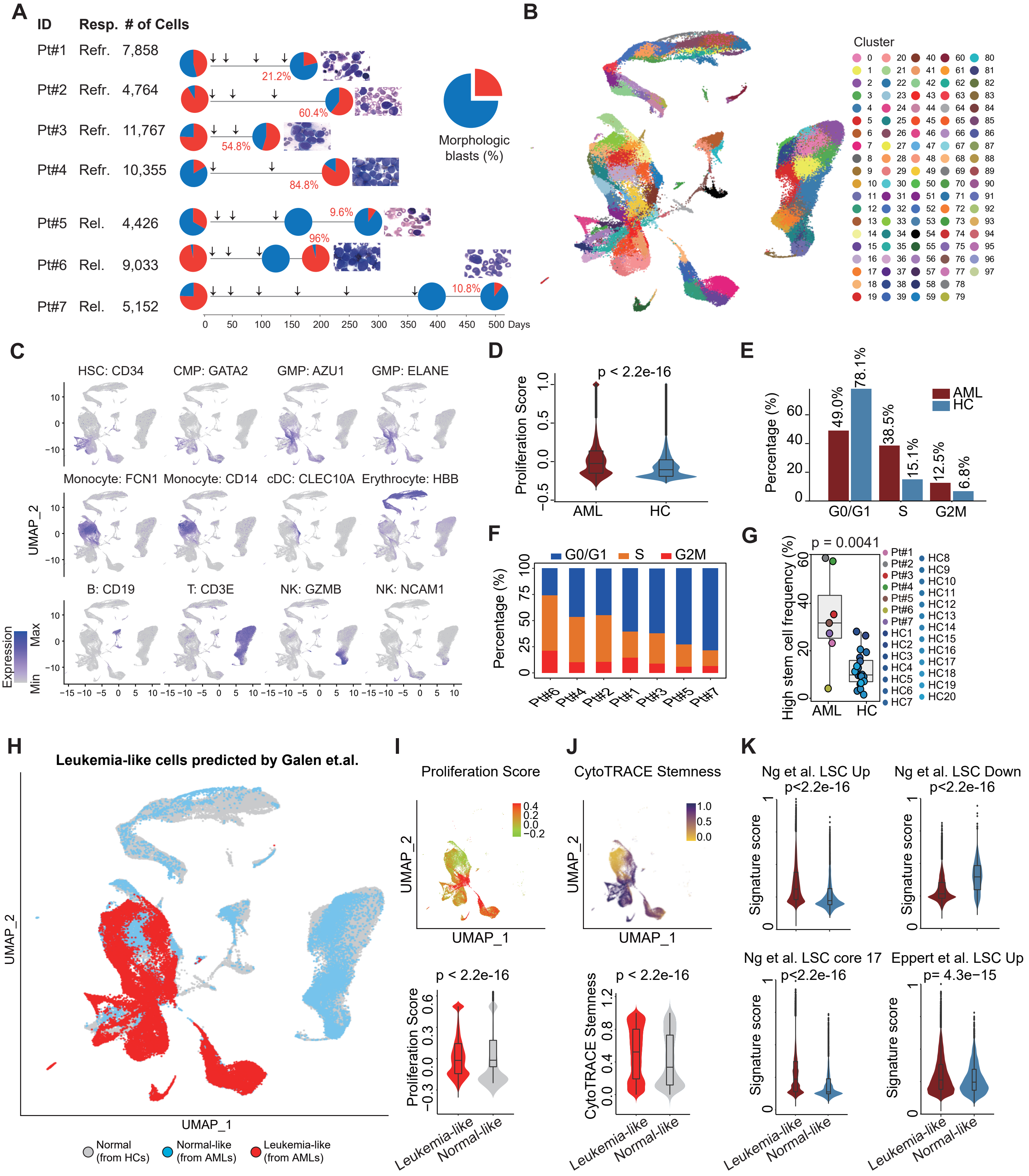

A
ID
Resp.
# of Cells
Pt#1 Refr. 7,858
Pt#2 Refr. 4,764
Pt#3 Refr. 11,767
Pt#4 Refr. 10,355
Pt#5 Rel. 4,426
Pt#6 Rel. 9,033
Pt#7 Rel. 5,152
21.2%
60.4%
54.8%
84.8%
9.6%
96%
10.8%
Morphologic blasts (%)
0 50 100 150 200 250 300 350 400 450 500 Days
B
Cluster
C
HSC: CD34
CMP: GATA2
GMP: AZU1
GMP: ELANE
Monocyte: FCN1
Monocyte: CD14
cDC: CLEC10A
Erythrocyte: HBB
B: CD19
T: CD3E
NK: GZMB
NK: NCAM1
UMAP_2
Expression
Max
Min
D
p < 2.2e-16
Proliferation Score
AML
HC
E
Percentage (%)
49.0%
78.1%
38.5%
15.1%
12.5%
6.8%
AML
HC
G0/G1
S
G2M
F
G0/G1
S
G2M
Percentage (%)
Pt#6 Pt#4 Pt#2 Pt#1 Pt#3 Pt#5 Pt#7
G
p = 0.0041
High stem cell frequency (%)
AML
HC
Pt#1 Pt#2 Pt#3 Pt#4 Pt#5 Pt#6 Pt#7
HC1 HC2 HC3 HC4 HC5 HC6 HC7 HC8 HC9 HC10 HC11 HC12 HC13 HC14 HC15 HC16 HC17 HC18 HC19 HC20
H
Leukemia-like cells predicted by Galen et.al.
UMAP_2
UMAP_1
Normal (from HCs)
Normal-like (from AMLs)
Leukemia-like (from AMLs)
I
Proliferation Score
UMAP_2
UMAP_1
p < 2.2e-16
Proliferation Score
Leukemia-like
Normal-like
J
CytoTRACE Stemness
UMAP_2
UMAP_1
p < 2.2e-16
CytoTRACE Stemness
Leukemia-like
Normal-like
K
Ng et al. LSC Up
p<2.2e-16
Ng et al. LSC Down
p<2.2e-16
Ng et al. LSC core 17
p<2.2e-16
Eppert et al. LSC Up
p= 4.3e−15
Signature score
Leukemia-like
Normal-like

Supplementary Figure S1. Unsupervised clustering and annotation of scRNA-seq data from bone marrow aspirates of RR-AML patients and healthy controls (HCs). (**A**) Summary of the four primary refractory and three short-term relapsed AML patients whose bone marrow aspirates were collected for scRNA-seq in this study. The black arrows indicate the time points of chemotherapy. Pie charts show the morphologic blasts percentage of bone marrow. (**B**) The unsupervised clustering of 118,565 cells. (**C**) Expression levels of known markers for specific hematopoietic cells overlaid on the UMAP representation. (**D**) Violin Plot showing the proliferation scores between cells from RR-AML patients and HCs. Horizontal lines in the boxplots represent the median, the lower and upper hinges correspond to the first and third quartiles, and the whiskers extend from the hinge up to 1.5 times the interquartile range from the hinge. (**E**) Percentage of cells in G1, S, and G2M phases from RR-AML patients and HCs. (**F**) Fraction of cells in G1 (blue), S (orange), and G2M (red) phases of each RR-AML patient. (**G**) Boxplot showing the percentage of cells with high stemness (CytoTRACE stemness > 0.75) in HCs and AML patients. Wilcoxon rank-sum test was used to measure the differences between groups. (**H**) UMAP visualization of the leukemia-like cells identified by classifier of Galen et al. Cells from HCs were shown in grey, normal-like cells from AML patients were shown in sky blue, and leukemia-like cells from AML patients were shown in red. (**I**) Upper: UMAP visualization of proliferation scores of cells from AML patients. Bottom: violin plot showing the proliferation scores between leukemia-like and normal-like cells in AML patients. Wilcoxon rank-sum test was used to measure the differences between groups. Erythrocytes and lymphocytes were excluded. (**J**) Upper: UMAP visualization of CytoTRACE stemness scores of cells from AML patients. Bottom: violin plot showing the CytoTRACE stemness scores between leukemia-like and normal-like cells in AML patients. Wilcoxon rank-sum test was used to measure the differences between groups. Erythrocytes and lymphocytes were excluded. (**K**) The LSC signature scores of leukemia-like and normal-like cells from AML patients. The LSC signatures were obtained from previous literatures, including LSC upregulated genes from Ng et al. and Eppert et al., 17 LSC core genes and LSC down-regulated genes from Ng et al.

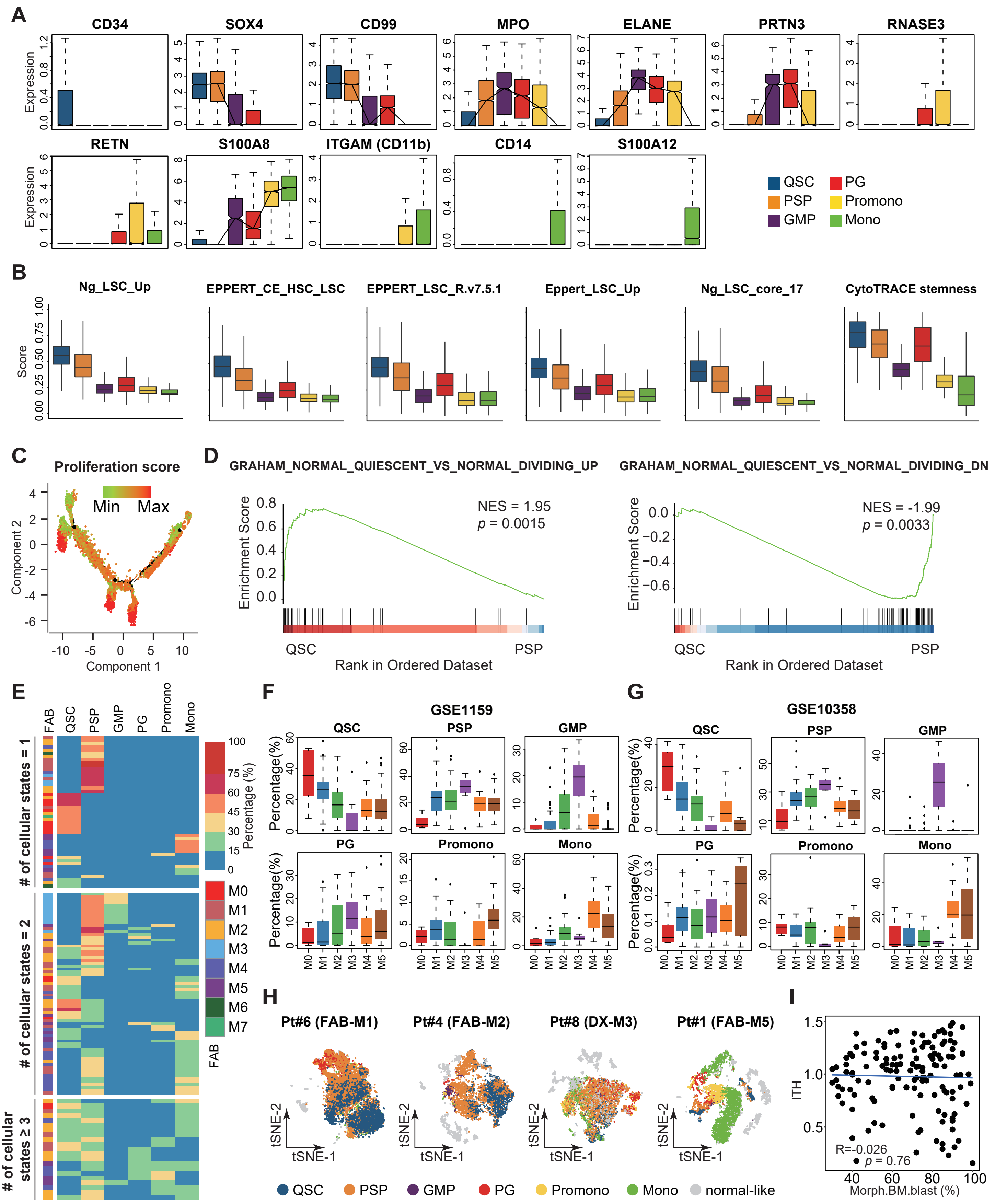
A
CD34
SOX4
CD99
MPO
ELANE
PRTN3
RNASE3
RETN
S100A8
ITGAM (CD11b)
CD14
S100A12
Expression
QSC
PSP
GMP
PG
Promono
Mono
B
Ng_LSC_Up
EPPERT_CE_HSC_LSC
EPPERT_LSC_R.v7.5.1
Eppert_LSC_Up
Ng_LSC_core_17
CytoTRACE stemness
Score
C
Proliferation score
Min
Max
Component 2
Component 1
D
GRAHAM_NORMAL_QUIESCENT_VS_NORMAL_DIVIDING_UP
GRAHAM_NORMAL_QUIESCENT_VS_NORMAL_DIVIDING_DN
NES = 1.95
p = 0.0015
NES = -1.99
p = 0.0033
Enrichment Score
Rank in Ordered Dataset
E
FAB
# of cellular states = 1
# of cellular states = 2
# of cellular states ≥ 3
Percentage (%)
M0
M1
M2
M3
M4
M5
M6
M7
F
GSE1159
G
GSE10358
Percentage(%)
H
Pt#6 (FAB-M1)
Pt#4 (FAB-M2)
Pt#8 (DX-M3)
Pt#1 (FAB-M5)
tSNE-1
tSNE-2
normal-like
I
ITH
R=-0.026
p = 0.76
Morph.BM.blast (%)

Supplementary Figure S2. Identification of leukemia-like cellular states and clinical relevance. (**A**) Boxplots showing the expression levels of established lineage-specific genes of six leukemia-like cellular states, including CD34, SOX4, and CD99 for QSCs and PSPs; MPO, ELANE, and PRTN3 for GMPs and PGs; RNASE3 and RETN for promono; S100A8, ITGAM(CD11b), CD14 and S100A12 for differentiated myeloid cells. (**B**) Boxplots showing the stemness signature scores among six cellular states of leukemia-like cells. (**C**) Proliferation scores of leukemia-like cells overlaid on the pseudotime trajectory. (**D**) GSEA plots showing the significant enrichment of quiescence signature in QSCs, and enrichment of proliferation signature in PSPs. (**E**) Heatmap showing the percentage of leukemia-like cellular states in each TCGA AML patient estimated by CIBERSORTx. Columns represent cellular states (QSC, PSP, GMP, PG, Promono, and Mono), and rows represent TCGA samples. Patients with only one cellular state > 15% were classified as “# of cellular states=1”, patients with two cellular states > 15% were classified as “# of cellular states=2”, and patients with more than two cellular states > 15% were classified as “# of cellular states ≥ 3”. (**F-G**) Boxplots showing the percentage of each cellular state in AML patients with different FAB subtypes from dataset GSE1159 (**F**) and GSE10358 (**G**). Horizontal lines in the boxplots represent the median, the lower and upper hinges correspond to the first and third quartiles, and the whiskers extend from the hinge up to 1.5 times the interquartile range from the hinge. (**H**) The cellular states of AML patients with different FAB subtypes showing at single-cell level. (**I**) Scatterplot showing the correlation between transcriptional ITH (y-axis) and morphologic blast percentage (x-axis) in TCGA AML cohort.

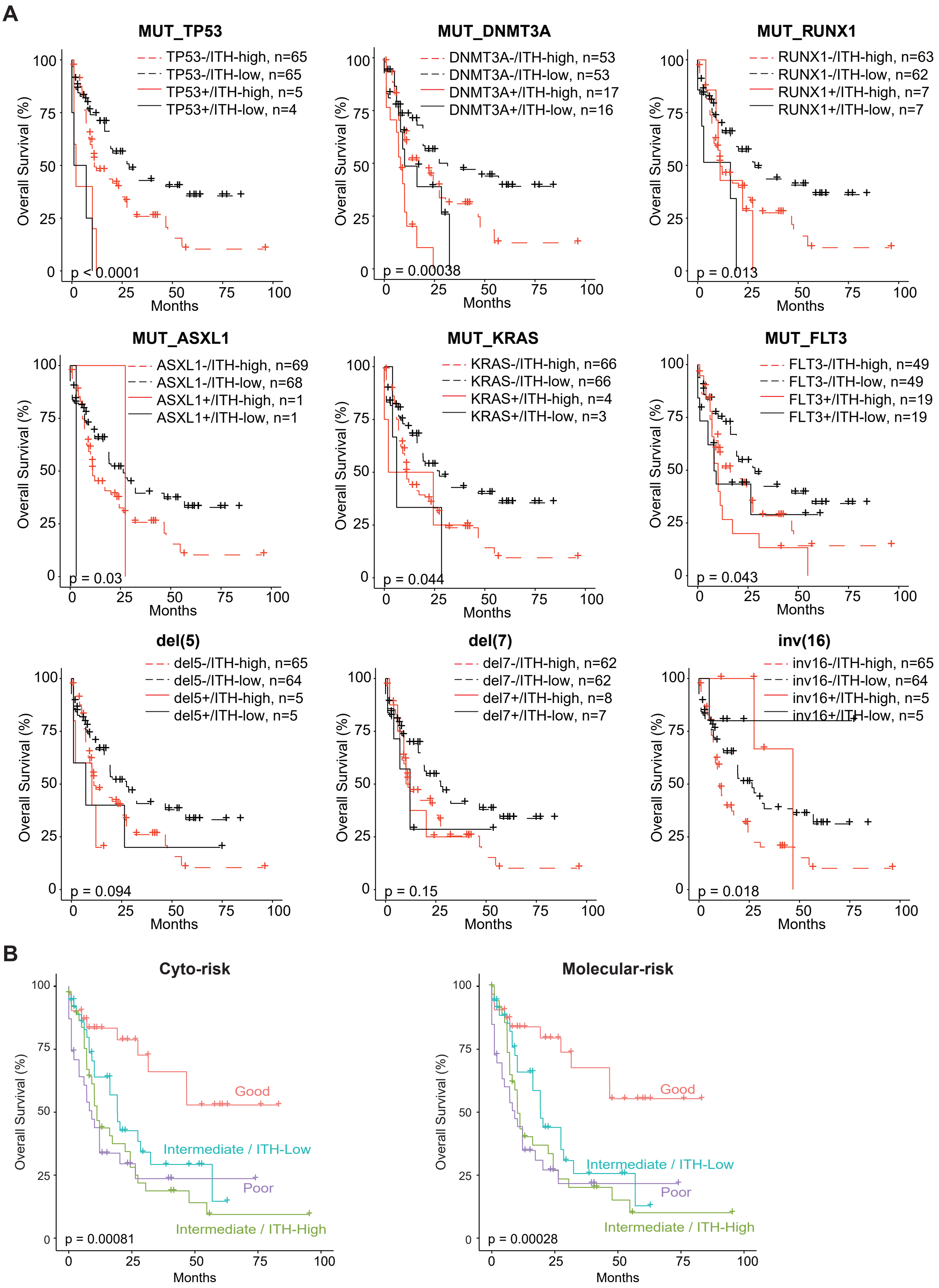

Supplementary Figure S3. Evaluations of the prognostic value of the transcriptomic ITH index for wild-type or intermediate risk AML patients. "+" indicates mutation and "-" indicates wild-type. Patients were categorized into two groups based on the median of ITH.

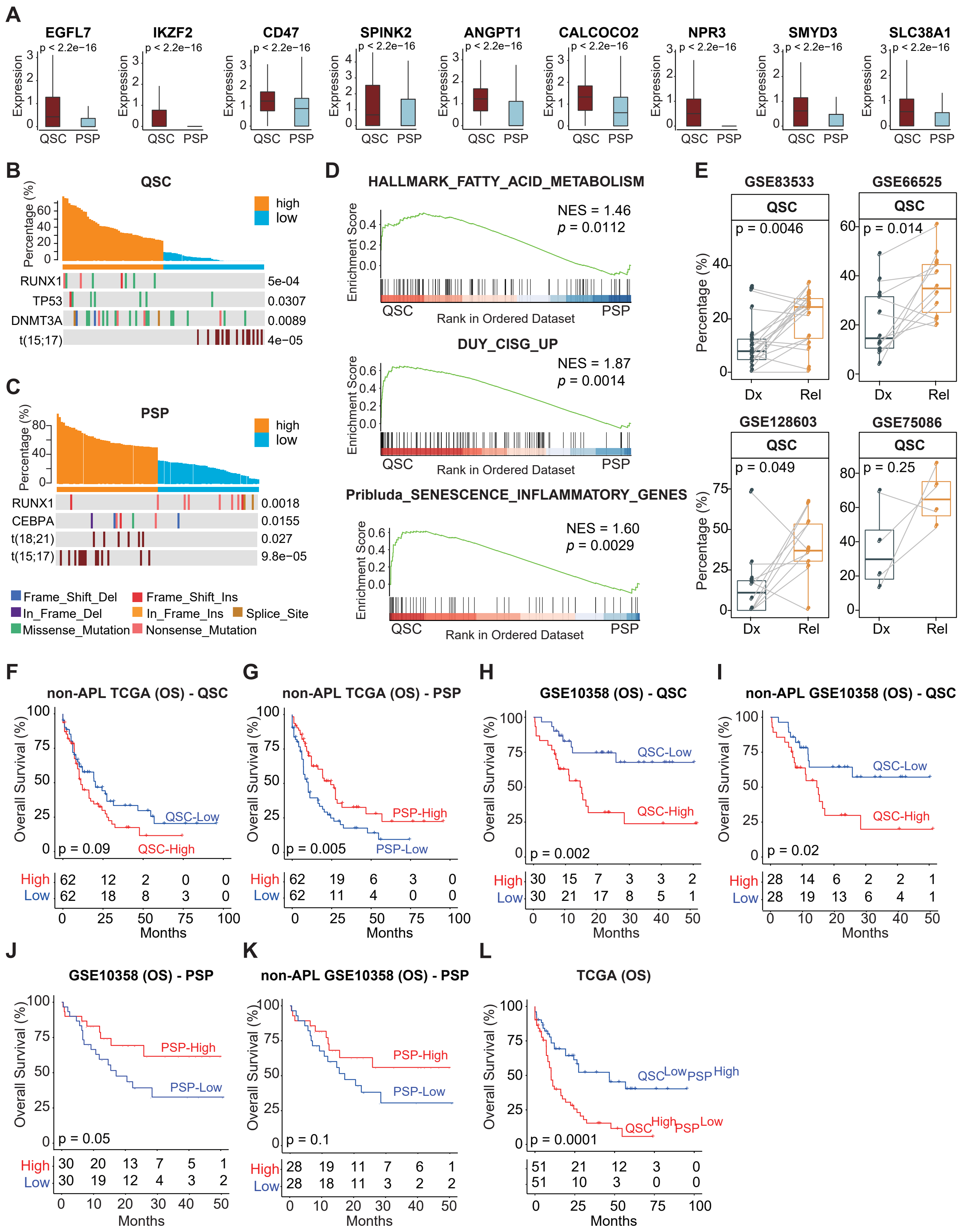
A
EGFL7
IKZF2
CD47
SPINK2
ANGPT1
CALCOCO2
NPR3
SMYD3
SLC38A1
p < 2.2e−16
Expression
QSC PSP
B
QSC
Percentage (%)
high
low
RUNX1 5e-04
TP53 0.0307
DNMT3A 0.0089
t(15;17) 4e-05
C
PSP
RUNX1 0.0018
CEBPA 0.0155
t(18;21) 0.027
t(15;17) 9.8e−05
Frame_Shift_Del
Frame_Shift_Ins
In_Frame_Del
In_Frame_Ins
Splice_Site
Missense_Mutation
Nonsense_Mutation
D
HALLMARK_FATTY_ACID_METABOLISM
NES = 1.46
p = 0.0112
DUY_CISG_UP
NES = 1.87
p = 0.0014
Pribluda_SENESCENCE_INFLAMMATORY_GENES
NES = 1.60
p = 0.0029
Enrichment Score
Rank in Ordered Dataset
E
GSE83533
p = 0.0046
GSE66525
p = 0.014
GSE128603
p = 0.049
GSE75086
p = 0.25
Dx Rel
F
non-APL TCGA (OS) - QSC
p = 0.09
QSC-Low
QSC-High
High 62 12 2 0 0
Low 62 18 8 3 0
G
non-APL TCGA (OS) - PSP
p = 0.005
PSP-High
PSP-Low
High 62 19 6 3 0
Low 62 11 4 0 0
H
GSE10358 (OS) - QSC
p = 0.002
High 30 15 7 3 3 2
Low 30 21 17 8 5 1
I
non-APL GSE10358 (OS) - QSC
p = 0.02
High 28 14 6 2 2 1
Low 28 19 13 6 4 1
J
GSE10358 (OS) - PSP
p = 0.05
High 30 20 13 7 5 1
Low 30 19 12 4 3 2
K
non-APL GSE10358 (OS) - PSP
p = 0.1
High 28 19 11 7 6 1
Low 28 18 11 3 2 2
L
TCGA (OS)
p = 0.0001
QSC Low PSP High
QSC High PSP Low
51 21 12 3 0
51 10 3 0 0
Overall Survival (%)
Months

Supplementary Figure S4. The correlation between QSCs/PSPs enrichment and genomic events, relapse, and survival. (**A**) Representative genes that highly expressed in QSCs compared with PSPs. (**B**) Relationship between QSCs abundance and genomic events in TCGA AML cohort. Fisher exact test was used to measure the differences between groups. (**C**) Relationship between PSPs abundance and genomic events in TCGA AML cohort. Fisher exact test was used to measure the differences between groups. (**D**) GSEA analysis of the differentially expressed genes between QSCs and PSPs in previously reported AML chemoresistance signatures. Results showed the significant upregulation of fatty acid metabolism and senescence-like signatures in the QSCs compared to PSPs. (**E**) Boxplots showing the proportion of QSCs and PSPs in matched diagnostic and relapsed patients. Paired Wilcoxon rank test was used to measure the differences between groups. (**F-G**) Kaplan-Meier analysis of OS of TCGA non-APL AML patients. All patients were categorized into two groups based on the median level of QSCs (**F**) or PSPs (**G**) percentage. (**H-K**) Kaplan-Meier analysis of OS of AML patients in GSE10358 dataset. (**L**) Kaplan-Meier analysis of OS of TCGA AML patients based on the level of QSCs and PSPs.

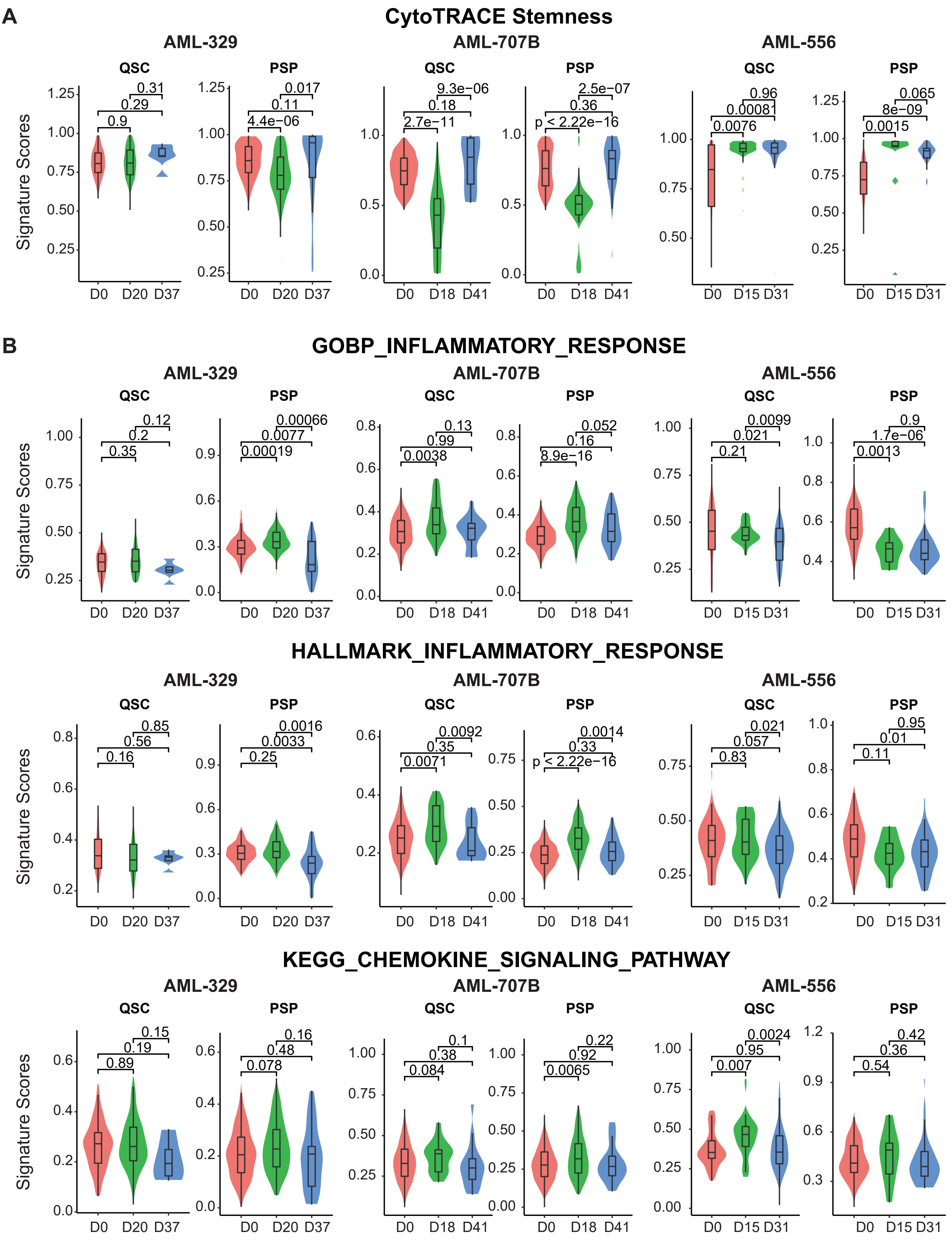

Supplementary Figure S5. Dynamic changes of stemness and inflammatory responses at different time points during chemotherapy.

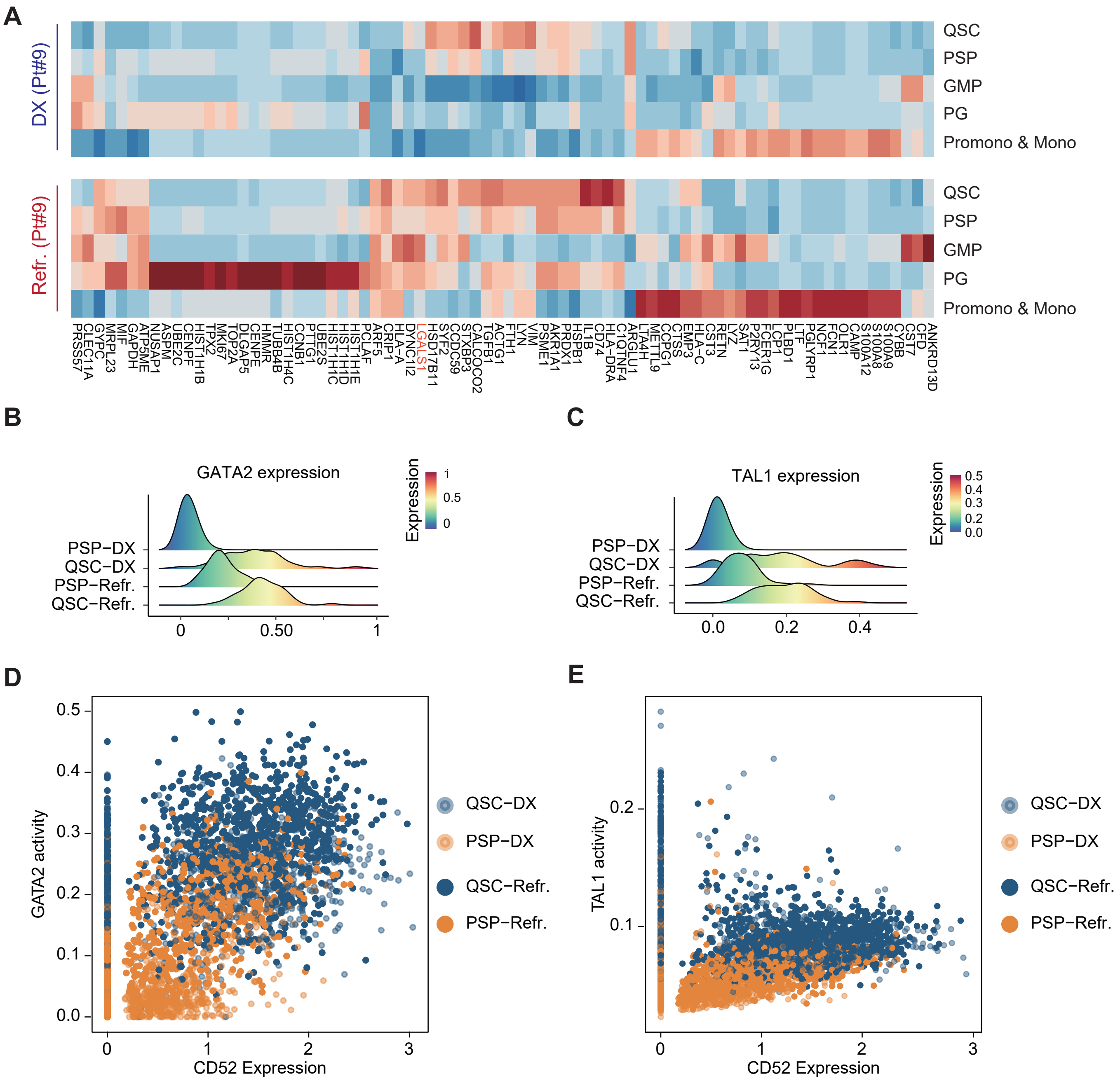

Supplementary Figure S6. The dynamic changes of transcription during chemotherapy in refractory AML patients. (**A**) Heatmap for differentially expressed genes after chemotherapy in Pt#9. (**B-C**) Ridge plots showing the expression levels of transcriptional factor GATA2 (**B**) and TAL1 (**C**) in QSCs and PSPs during chemotherapy. (**D-E**) Scatter plots showing the correlation between CD52 expression and GATA2 (**D**) and TAL1 (**E**) activity.

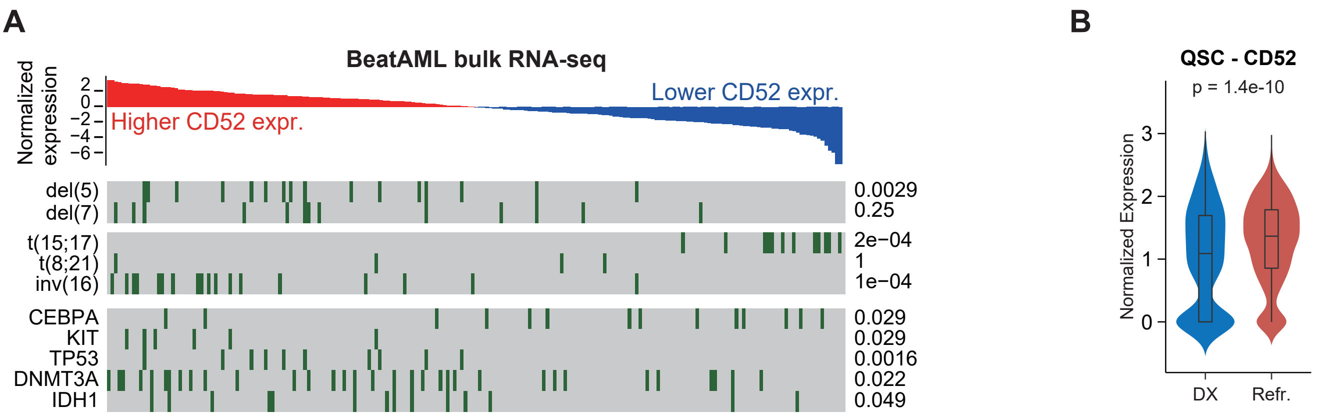

Supplementary Figure S7. The functional implications of CD52-SIGLEC10 interaction in AML patients. (**A**) Relationship between CD52 expression levels and genomic events in TCGA AML cohort. Fisher exact test was used to measure the differences between groups. (**B**) The expression level of CD52 in the QSC cells of Pt#3 before and after chemotherapy. Wilcoxon rank-sum test was used to measure the differences between groups.

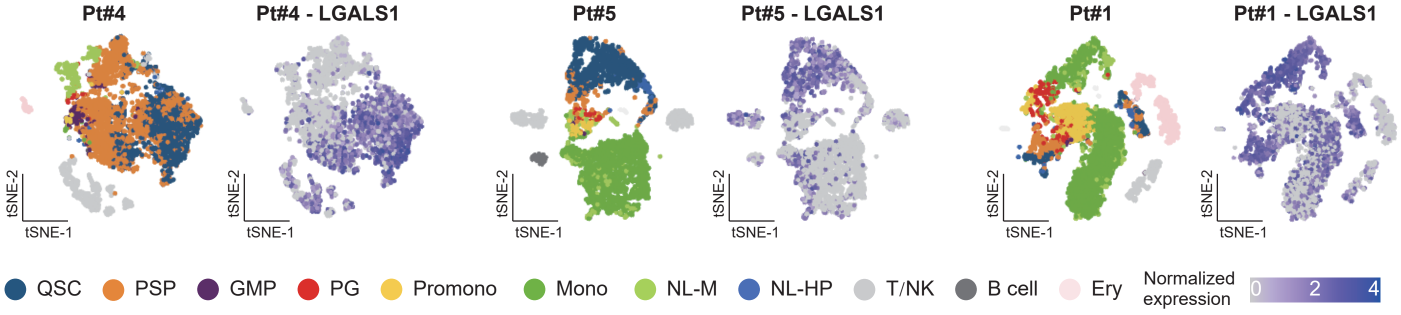

Supplementary Figure S8. The expression of LGALS1 in AML patients Pt#1, Pt#4, and Pt#5 at single-cell level.

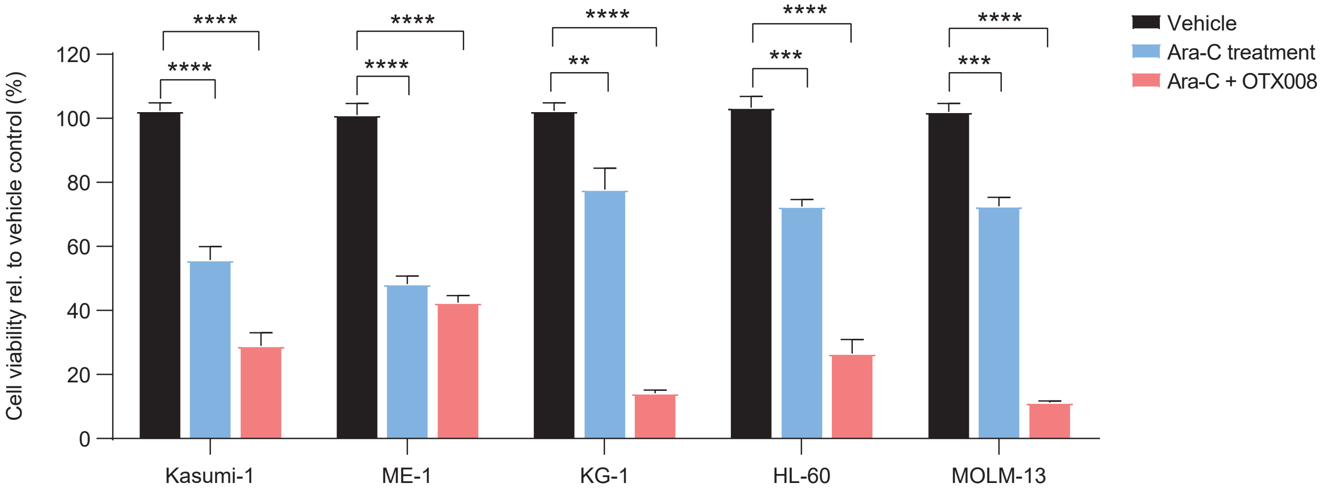

Supplementary Figure S9. CCK-8 assay showing the cell viability from five AML cell lines treated with DMSO, Cytarabine (200nm), and Cytarabine (200nm) + OTX008 (30 μM) combination for 24h.

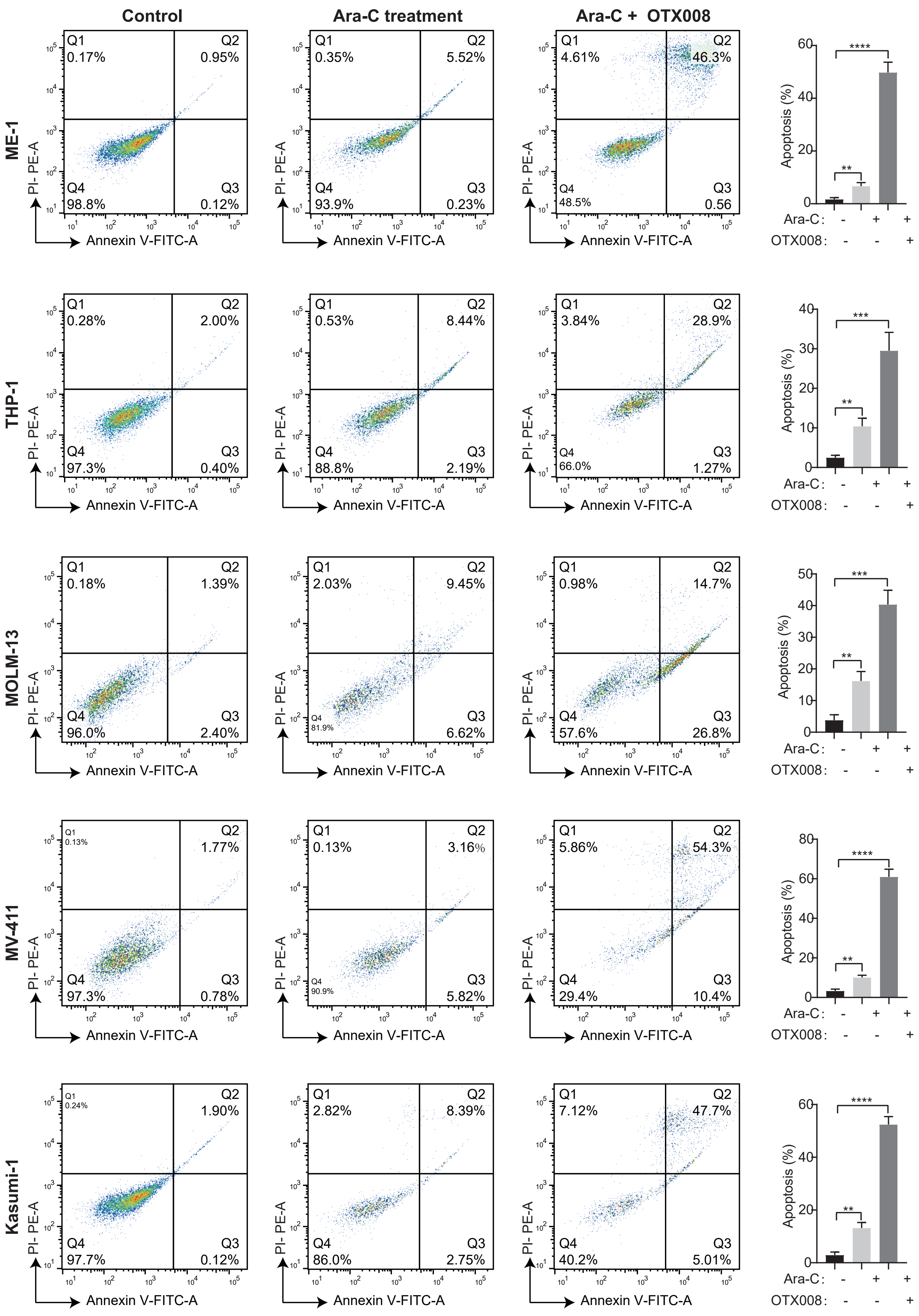
Control
Ara-C treatment
Ara-C + OTX008
ME-1
Q1 0.17%
Q2 0.95%
Q4 98.8%
Q3 0.12%
Q1 0.35%
Q2 5.52%
Q4 93.9%
Q3 0.23%
Q1 4.61%
Q2 46.3%
Q4 48.5%
Q3 0.56
THP-1
Q1 0.28%
Q2 2.00%
Q4 97.3%
Q3 0.40%
Q1 0.53%
Q2 8.44%
Q4 88.8%
Q3 2.19%
Q1 3.84%
Q2 28.9%
Q4 66.0%
Q3 1.27%
MOLM-13
Q1 0.18%
Q2 1.39%
Q4 96.0%
Q3 2.40%
Q1 2.03%
Q2 9.45%
Q4 81.9%
Q3 6.62%
Q1 0.98%
Q2 14.7%
Q4 57.6%
Q3 26.8%
MV-411
Q1 0.13%
Q2 1.77%
Q4 97.3%
Q3 0.78%
Q1 0.13%
Q2 3.16%
Q4 90.9%
Q3 5.82%
Q1 5.86%
Q2 54.3%
Q4 29.4%
Q3 10.4%
Kasumi-1
Q1 0.24%
Q2 1.90%
Q4 97.7%
Q3 0.12%
Q1 2.82%
Q2 8.39%
Q4 86.0%
Q3 2.75%
Q1 7.12%
Q2 47.7%
Q4 40.2%
Q3 5.01%
PI- PE-A
Annexin V-FITC-A
Apoptosis (%)
Ara-C: - + +
OTX008: - - +
**
***
****

Supplementary Figure S10. Flow cytometric analysis for cell apoptosis of different AML cell lines with different treatment strategies. Five AML cell lines were treated with Ara-C and OTX008 alone or in combination for 24h. Representative results are shown on the left and statistical results are shown on the right.

A

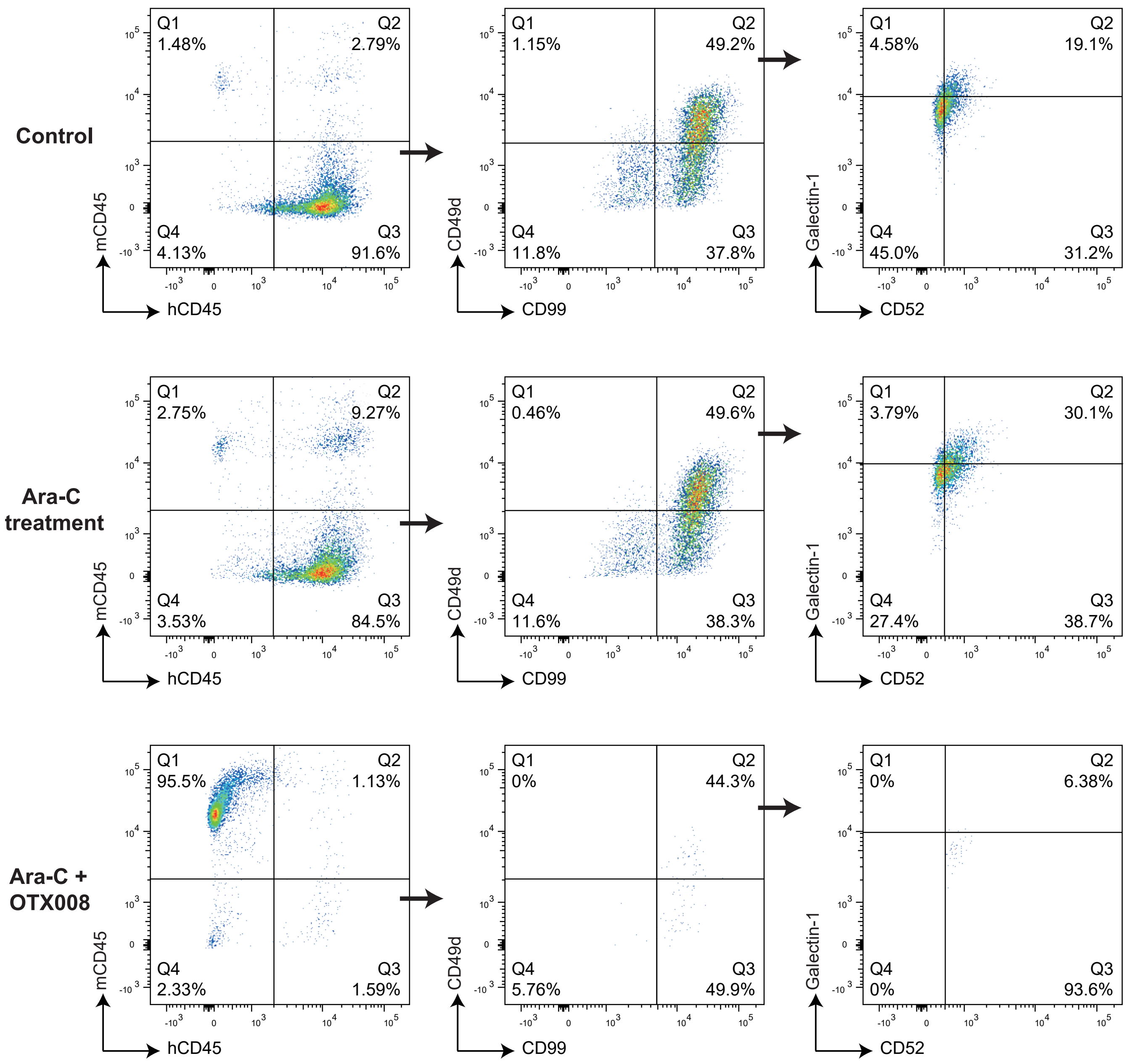
Control
Ara-C treatment
Ara-C + OTX008
Q1 1.48%
Q2 2.79%
Q4 4.13%
Q3 91.6%
Q1 1.15%
Q2 49.2%
Q4 11.8%
Q3 37.8%
Q1 4.58%
Q2 19.1%
Q4 45.0%
Q3 31.2%
Q1 2.75%
Q2 9.27%
Q4 3.53%
Q3 84.5%
Q1 0.46%
Q2 49.6%
Q4 11.6%
Q3 38.3%
Q1 3.79%
Q2 30.1%
Q4 27.4%
Q3 38.7%
Q1 95.5%
Q2 1.13%
Q4 2.33%
Q3 1.59%
Q1 0%
Q2 44.3%
Q4 5.76%
Q3 49.9%
Q1 0%
Q2 6.38%
Q4 0%
Q3 93.6%
mCD45
hCD45
CD49d
CD99
Galectin-1
CD52

B

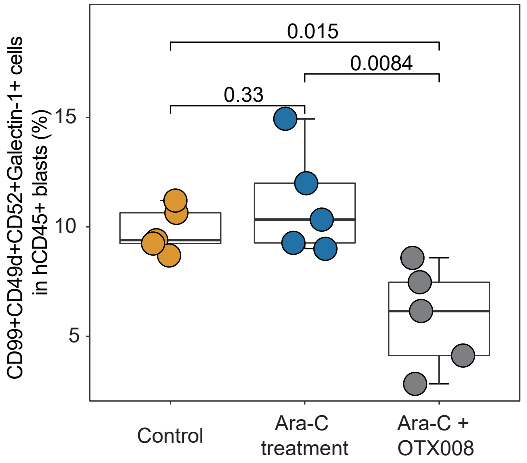
CD99+CD49d+CD52+Galectin-1+ cells in hCD45+ blasts (%)
0.015
0.0084
0.33
15
10
5
Control
Ara-C treatment
Ara-C + OTX008

Supplementary Figure S11. Flow cytometric analysis of QSCs in bone marrows from AML xenograft model of different treatment groups. (**A**) Representative flow cytometric analysis of the percentage of CD99+CD49d+CD52+-Galectin-1+ (QSCs) cells in bone marrow samples from AML xenograft model of different treatment groups. (**B**) Quantification of CD99+CD49d+CD52+Galectin-1+ (QSCs) cells in different treatment groups.
