# Supplementary material for: Single-cell analysis reveals the chemotherapy-induced cellular reprogramming and novel therapeutic targets in relapsed/refractory acute myeloid leukemia
Source: Leukemia. 2022 Dec 21;37(2):308–25. doi: 10.1038/s41375-022-01789-6 (PMC9898038; doi:10.1038/s41375-022-01789-6)
Supplement: Supplementary file 3 — Supplementary Methods [file 41375_2022_1789_MOESM3_ESM.docx]

**Supplementary Methods**

**Alignment and pre-processing procedures of scRNA-Seq data**

Single-cell library sequencing was performed on Illumina HiSeq XTen, with 150 bp paired-end sequencing. Cell Ranger 3.0.2 was used to perform sample demultiplexing, barcode processing, and generating gene count data for each cell. The cDNA insert was aligned to the hg38/GRCh38 reference genome. The feature-barcode matrices were generated for each sample by counting the valid barcodes and unique molecular identifiers (UMIs). Further analyses, including quality control, identification of highly variable features, and unsupervised clustering, were performed using Seurat (3.1.5, <http://satijalab.org/seurat/>) R toolkit^1^. To eliminate the influence of abnormal cells and technical noise on downstream analysis, we removed the low-quality cells, including doublets and empty droplets. Cells were removed if the expression of mitochondrial genes was greater than 10% or with detected genes less than 200 or greater than 6,000. The number of cells before and after filtering for each sample was listed in **Supplementary Table S1**. Considering the similarities of differentiation states between leukemia and normal hematological cells in the bone marrow ecosystem, we downloaded data from Oetjen et al.^2^, a scRNA-seq dataset of the bone marrow specimens of 20 healthy donors as references. The quality control and pre-processing procedures were as the same with in-house data.

**Data integration**

The Seurat anchor-based method was used to integrate multiple single-cell datasets to minimize the technical batch effects among individuals and experiments. The integration anchors were identified based on the first 30 dimensions from canonical correlation analysis (CCA) by using the “FindIntegratedAnchors” function. The combined datasets included 118,565 cells from 27 samples.

**Clustering, visualization, and cell-type annotation**

Standard procedures for scaling integrated data matrix, dimensionality reduction, clustering, and visualization were performed using the R package Seurat v3^1^. The top 4,000 variable genes of scaled data were used for principal component analysis (PCA) to reduce dimensionality. For the integrated data with 118,565 cells from 27 samples, the dimensionality of the scaled integrated data matrix was further reduced to two-dimensional space based on the first 35 principal components (PCs) and visualized by uniform manifold approximation and projection (UMAP). The cell clusters were identified based on a shared nearest neighbor (SNN) modularity optimization-based clustering algorithm with a resolution of 5, and all cells were divided into 98 clusters. Hierarchical clustering results showed that 98 clusters could be categorized into four groups. In order to recognize the types of these cells, we further used SingleR to annotate cell types based on the NovershternHematopoieticData database^3,4^. Besides, some known markers for specific hematopoietic cells, such as CD34 for HSCs, CD19 for B cells, and CD3E for T cells, were used to verify the annotation of cell types.

**Evaluation of proliferation and stemness for each single cell**

To evaluate the cell cycle state of every single cell, we used the “CellCycleScoring” function in the Seurat package to calculate the signature score of a series of cell cycle-related markers, including 43 genes associated with the S phase and 54 genes associated with the G2M phase. Besides, we also evaluated the proliferation score for every cell by using a signature of ten highly expressed genes in cycling cells (ASPM, CENPE, CENPF, DLGAP5, MKI67, NUSAP1, PCLAF, STMN1, TOP2A, TUBB). Using the method of previous study^5^, we selected the 100 genes with the most similar average expression levels as a background gene set for each of these genes. The average expression of the background gene set was then subtracted from the respective signature gene, and the average of the resulting values of all signature genes was defined as the cell-cycle score. In addition, we calculated the stemness score for each single-cell by using CytoTRACE^6^, an established computational framework for stemness evaluation based on transcriptional diversity. Furthermore, to evaluate the reliability of identified leukemia-like and normal-like cells in AML patients, we calculated the LSC score with a series of gene signatures from Ng et al.^7^ and Eppert et al.^8^ by utilizing single-sample gene sets enrichment analysis (ssGSEA)^9^.

**Characterization of the differentiation trajectory of leukemia-like cellular states**

Considering cells from healthy donors account for ~ 60% of all cells, we defined clusters as normal-like if the percentage of cells from healthy donors was above 60%. AML single cells belonging to these clusters were classified as normal-like, and the remaining cells were classified as leukemia-like. In order to analyze the cellular states of leukemia-like cells, we used R package Monocle2 (version 2.14.0) to characterize the differentiation trajectory of the identified leukemia-like cells^10,11^. Cellular states were identified based on the pseudotime analysis. Using the well-known gene markers of various hematopoietic development stages, we fitted the expression change of lineage-specific genes along with the pseudotime trajectory based on Locally Weighted Regression (LOESS). Finally, we defined six cellular states of leukemia-like cells based on the differentiation stages, proliferation, and stemness score, including QSC, PSP, GMP, PG, promono, and mono.

**Identification of specifically expressed genes in each cellular state**

We identified genes specifically expressed in each leukemia-like cellular state using the "FindMarkers" function of the Seurat package. Genes with log2 (fold-change) greater than 0.25 and at least expressed in 25% of cells were identified as specific genes of each cellular state. Furthermore, combined with the information in public databases Cell Surface Protein Atlas (CSPA, http://wlab.ethz.ch/cspa/)^12^, Gene Ontology (GO, http://geneontology.org/), and data from Fluck et al.^13^, we identified the specific surface markers of each cellular state. Cell Surface markers with a percentage ratio greater than 1.5 were shown in Figure 2E. In addition, to compare the transcriptional patterns between QSC and PSP more strictly, we took the expression percentage of genes (perc_genes) in each cellular state into consideration and calculated the ratio of perc_genes between two groups (perc_ratio). Then, genes with avg_logFC≥0.5 and perc_ratio≥2 were selected and shown in Figure 3A.

**Differential regulatory activity analysis**

Transcriptional regulon analysis was performed on six cellular states by utilizing pySCENIC^14^, which was a computational tool that identified transcriptional regulons and assessed the activity score of regulons in the individual cell. Default parameters were used. We identified the regulons which were differentially activated in QSC and PSP cells using Wilcoxon rank-sum test. Fold-changes (FC) of activity score and p-values were measured. In addition, we calculated the expression percentage of each TF (perc_TF) in QSC and PSP cells, respectively. Regulons with perc_TF>5%, FC>1.3 and p-values＜1e-12 were identified as differentially activated regulons.

**Single-cell metabolic pathway analysis**

The single-cell metabolic pathway analysis was conducted by the method from Xiao et al.^15^, which could calculate the ssGSEA scores of 85 metabolic pathways of [Kyoto Encyclopedia of Genes and Genomes](https://www.genome.jp/kegg/) (KEGG) based on the gene expression in every single cell. The leukemia-like cells were divided into 42 clusters by unsupervised clustering. We calculated the metabolic pathway scores for each cluster to recognize the differentially activated pathways between QSC and PSP cells. Clusters were defined as the cellular state with the largest proportion. Differentially activated metabolic pathways were identified by Wilcoxon rank-sum test. Pathways with p < 0.05 in QSC clusters were shown.

**Deconvolution of bulk transcriptomic data**

In order to explore the clinical relevance of each leukemia-like cellular state, we estimated the cell type abundances of bulk transcriptomic data from AML patients or cultured cells by using CIBERSORTx^16^. Single cells in this study were used as a reference and labeled as different cell types. Because of the limitation of input size for CIBERSORTx, single cells were randomly selected as input. For each cellular state, half were randomly selected if the number of cells was less than 1,500, one-third for the others. For normal cell types (B cells, NK/T cells, erythrocytes, HSC/Progenitors, and myeloid cells), 500 cells were randomly selected. Only leukemia-like cells were included as input references for the estimation of bulk datasets from in-vitro cultured AML cells. Both normal and leukemia-like cells were included as input references to estimate bulk datasets from AML patients.

We downloaded the bulk RNA-seq data of samples from 151 AML patients of the TCGA-LAML cohort from the UCSC Xena (<https://xenabrowser.net/datapages/>). Samples without available survival or FAB-subtypes information were further excluded. Finally, we obtained the transcriptomic and clinical data of 139 AML patients. Based on the estimated percentage of different cellular states, we compared the relative abundances of each cellular state in AML patients with various FAB subtypes. Besides, Shannon entropy was used to calculate the ITH of each TCGA AML patient. Considering the similarity of signature genes between PSP and GMP, we added up the percentages of these cells when calculating Shannon entropy.

**Evaluation of the association between genomic and transcriptional ITH**

The number of AML-related gene mutations was counted for each patient in TCGA dataset, including FLT3, NPM1, DNMT3A, IDH1, IDH2, RUNX1, TET2, TP53, NRAS, CEBPA, KIT, KRAS, PHF6 and ASXL1. Next, we performed dimensionality reduction according to the expression values of the top 100 most variable genes by using R package “Rtsne” (Figure 2H). Then, we compared the transcriptional ITH scores among patients with different number of AML-related gene mutations to explore the correlation between genomic and transcriptional ITH.

**Cell-cell communication analysis**

We used the CellPhoneDB^17^ to identify the interactions between different cell types. The ligand-receptor interactions with a p-value＜0.05 were selected. To further analyze the functional implication of CD52-SIGLEC10 interaction in AML patients, we calculated the combined expression of CD52 and SIGLEC10 in the TCGA AML cohort by utilizing the ssGSEA method. Besides, the scores of 50 hallmarks from the MSigDB database^18^ of each patient were also calculated based on ssGSEA^9^. The Pearson's correlation between CD52-SIGLEC10 combination score and each hallmark was measured by the R function "cor.test".

**Analysis of the dynamic cellular and transcriptional changes after chemotherapy**

Using the defined cellular states as a reference, we annotated the single cells from longitudinal matched samples by transform function in the Seurat package by using the "FindTransferAnchors" and "TransferData" function. Both the in-house and matched samples from Galen et al.^5^ were annotated to trace the cellular programming induced bu chemotherapy. The cellular changes after chemotherapy were described by the Sankey diagram. Specifically, for the data from Galen et al.^5^, only patients who received chemotherapy and with >500 cells at each timepoint were selected for analysis, including patient AML556, AML329, and AML707B. In order to further understand the transcriptional changes after chemotherapy in refractory patients, we identified the differentially expressed genes between the diagnostic and treated samples for each cellular state. Furthermore, based on the information in GENECODE database, we selected the protein-coding genes with the difference of expression percentage above 0.2. Next, we calculated the average expression values of each cellular state at each timepoint. The top 15 genes with the highest log_avgFC in each cellular state were shown.

In order to investigate the dynamic changes in transcriptional regulations during chemotherapy treatment and identified the potential regulatory factors that involved in the reprogramming of PSP cells, we utilized the pySCENIC^14^ to calculate the regulatory activity scores of transcription factors in refractory patients. Both the activity scores and expression values were compared among QSC-DX, PSP-DX, PSP-Refr. and PSP-refr. group. All cells in each group were divided into 50 clusters randomly, and the distribution of the average values were shown in ridge plots. The CHIP-seq data of GATA2 and TAL1 in K562 cell line were downloaded from GSE170040 and GSE91490, respectively. The CHIP-seq peaks were visualized by IGV tools^19^.

**Survival Analysis**

Kaplan-Meier survival analysis was performed by the “ggsurvplot” function in the R package survminer. The log-rank test was used to evaluate the survival differences between groups.

**References**

1 Butler, A., Hoffman, P., Smibert, P., Papalexi, E. & Satija, R. Integrating single-cell transcriptomic data across different conditions, technologies, and species. *Nat Biotechnol* **36**, 411-420, doi:10.1038/nbt.4096 (2018).

2 Oetjen, K. A. *et al.* Human bone marrow assessment by single-cell RNA sequencing, mass cytometry, and flow cytometry. *JCI Insight* **3**, doi:10.1172/jci.insight.124928 (2018).

3 Novershtern, N. *et al.* Densely interconnected transcriptional circuits control cell states in human hematopoiesis. *Cell* **144**, 296-309, doi:10.1016/j.cell.2011.01.004 (2011).

4 Aran, D. *et al.* Reference-based analysis of lung single-cell sequencing reveals a transitional profibrotic macrophage. *Nat Immunol* **20**, 163-172, doi:10.1038/s41590-018-0276-y (2019).

5 van Galen, P. *et al.* Single-Cell RNA-Seq Reveals AML Hierarchies Relevant to Disease Progression and Immunity. *Cell* **176**, 1265-1281 e1224, doi:10.1016/j.cell.2019.01.031 (2019).

6 Gulati, G. S. *et al.* Single-cell transcriptional diversity is a hallmark of developmental potential. *Science* **367**, 405-411, doi:10.1126/science.aax0249 (2020).

7 Ng, S. W. *et al.* A 17-gene stemness score for rapid determination of risk in acute leukaemia. *Nature* **540**, 433-437, doi:10.1038/nature20598 (2016).

8 Eppert, K. *et al.* Stem cell gene expression programs influence clinical outcome in human leukemia. *Nat Med* **17**, 1086-1093, doi:10.1038/nm.2415 (2011).

9 Subramanian, A. *et al.* Gene set enrichment analysis: a knowledge-based approach for interpreting genome-wide expression profiles. *Proc Natl Acad Sci U S A* **102**, 15545-15550, doi:10.1073/pnas.0506580102 (2005).

10 Trapnell, C. *et al.* The dynamics and regulators of cell fate decisions are revealed by pseudotemporal ordering of single cells. *Nat Biotechnol* **32**, 381-386, doi:10.1038/nbt.2859 (2014).

11 Qiu, X. *et al.* Single-cell mRNA quantification and differential analysis with Census. *Nat Methods* **14**, 309-315, doi:10.1038/nmeth.4150 (2017).

12 Bausch-Fluck, D. *et al.* A mass spectrometric-derived cell surface protein atlas. *PLoS One* **10**, e0121314, doi:10.1371/journal.pone.0121314 (2015).

13 Bausch-Fluck, D. *et al.* The in silico human surfaceome. *Proc Natl Acad Sci U S A* **115**, E10988-E10997, doi:10.1073/pnas.1808790115 (2018).

14 Aibar, S. *et al.* SCENIC: single-cell regulatory network inference and clustering. *Nat Methods* **14**, 1083-1086, doi:10.1038/nmeth.4463 (2017).

15 Xiao, Z., Dai, Z. & Locasale, J. W. Metabolic landscape of the tumor microenvironment at single cell resolution. *Nat Commun* **10**, 3763, doi:10.1038/s41467-019-11738-0 (2019).

16 Newman, A. M. *et al.* Determining cell type abundance and expression from bulk tissues with digital cytometry. *Nat Biotechnol* **37**, 773-782, doi:10.1038/s41587-019-0114-2 (2019).

17 Efremova, M., Vento-Tormo, M., Teichmann, S. A. & Vento-Tormo, R. CellPhoneDB: inferring cell-cell communication from combined expression of multi-subunit ligand-receptor complexes. *Nat Protoc* **15**, 1484-1506, doi:10.1038/s41596-020-0292-x (2020).

18 Liberzon, A. *et al.* The Molecular Signatures Database (MSigDB) hallmark gene set collection. *Cell Syst* **1**, 417-425, doi:10.1016/j.cels.2015.12.004 (2015).

19 Robinson, J. T. *et al.* Integrative genomics viewer. *Nat Biotechnol* **29**, 24-26, doi:10.1038/nbt.1754 (2011).
